# ZNFX1 functions as a compensatory dsRNA recognition receptor to exert antiviral effect in orange-spotted grouper

**DOI:** 10.1371/journal.ppat.1013652

**Published:** 2025-10-29

**Authors:** Rui Zheng, Siyou Huang, Yiling Kang, Jie Gao, Bingya Wu, Qijin Miao, Jianguo He, Junfeng Xie

**Affiliations:** State Key Laboratory of Biocontrol, Southern Marine Science and Engineering Guangdong Laboratory (Zhuhai), China-ASEAN Belt and Road Joint Laboratory on Mariculture Technology, Guangdong Provincial Key Laboratory of Aquatic Economic Animals, School of Life Sciences, Sun Yat-sen University, Guangzhou, Guangdong, China; CSIRO ACDP: CSIRO Australian Centre for Disease Preparedness Business Unit, AUSTRALIA

## Abstract

Zinc finger NFX1-type containing 1 (ZNFX1) has been established as a critical mediator of the antiviral response in mammals, functioning through dsRNA recognition and priority activation of the retinoic acid-inducible gene I (RIG-I)-like receptor (RLR) signaling pathway. However, the role of its fish ortholog, particularly in relation to aquatic virus interactions, remains elusive. The absence of the RIG-I homolog, a key pattern recognition receptor, in multiple Actinopterygii may compromise their innate antiviral immune responses. Here, ZNFX1 in *Epinephelus coioides* (EcZNFX1) is identified as an evolutionarily primitive, interferon (IFN)-stimulated dsRNA sensor that compensates for the absence of RIG-I in Actinopterygii. EcZNFX1 is rapidly upregulated by orange-spotted grouper nervous necrosis virus (OGNNV) infection and restricts viral replication in grouper brain-derived (GB) and spleen-derived (GS) cells after binding to viral dsRNA intermediates via its conserved P-loop NTPase domain. Notably, EcZNFX1 exerts a dual immunoregulatory role in modulating virus-induced inflammatory responses in diverse cellular contexts. In GB cells that are highly susceptible to OGNNV and have attenuated regenerative capacity, EcZNFX1 suppresses IFN-I/ISGs production and pyroptosis mediated by viral infection, thereby limiting neurotoxicity by precise tuning of the antiviral response. Conversely, in GS cells with stronger resistance to OGNNV, EcZNFX1 directly interacts with TBK1 to promote its phosphorylation and subsequent nuclear translocation of IRF3, activating a robust IFN-I signaling. Overall, this study elucidates that ZNFX1 is a compensatory receptor for dsRNA sensing in RIG-I-deficient teleost, which exerts context-dependent antiviral effects in cell-type-specific microenvironments, providing mechanistic insights for aquatic virus countermeasures.

## Authors summary

A diverse array of fish in Actinopterygii lack the canonical dsRNA receptor RIG-I. Whether this deficiency undermines the capacity of fish to defend against RNA viruses, whether alternative antiviral dsRNA receptors exist, and how the intricate interplay between the fish immune system and aquatic viruses operates remain unresolved questions. In this study, EcZNFX1 has been identified as a compensatory dsRNA sensor that is rapidly upregulated upon OGNNV infection. By binding viral dsRNA, it exerts dual immunoregulatory functions in diverse cellular contexts to modulate virus-induced inflammatory responses, ultimately inhibiting viral replication. Mechanistically, in brain cells, it attenuates neurotoxicity by suppressing IFN-I and pyroptosis, whereas in spleen cells, it robustly activates TBK1-dependent IFN-I signaling. Our study highlights EcZNFX1 as a compensatory antiviral dsRNA receptor in RIG-I-deficient fish species. It provides insights for developing strategies against NNV and offers novel perspectives for investigating the antiviral innate immune mechanisms in Actinopterygii lacking RIG-I.

## Introduction

Although osteichthyes are the earliest vertebrate lineage to develop adaptive immunity, their immune systems are generally considered to be in the “primary stage” [1]. The larvae and juveniles of osteichthyes mainly rely on innate immunity to defend against pathogen invasion, due to the incomplete development and functional immaturity of their adaptive immune systems. As a rapid response mechanism of the immune system, the innate immune system relies on a variety of pattern recognition receptors (PRRs) to recognize pathogen-associated molecular patterns (PAMPs), and recruits adaptors to establish an immune barrier [2]. Prior to the establishment of specific adaptive immune responses, the rapidly induced innate immune response is crucial for preventing viral replication and dissemination. The retinoic acid–inducible gene I (RIG-I)–like receptors-mediated signaling pathway is essential for the host’s antiviral defense against RNA virus infection. RLRs including RIG-I and melanoma differentiation-associated gene 5 (MDA5) from Helicase superfamily 2 (SF2) can recognize viral-derived dsRNA through the helicase domain [3]. The caspase activation and recruitment domains (CARDs) of RIG-I and MDA5 proteins interact with mitochondrial antiviral signalling protein (MAVS), which subsequently activates TANK binding kinase 1 (TBK1) [4]. TBK1 then phosphorylates transcription factors interferon regulatory factor 3 and 7 (IRF3 and IRF7), thereby IRF3 and IRF7 translocate into the nucleus and initiate the transcription of type I interferon (IFN-I) [5]. IFN-I of teleost fish can be recognized and bound by receptors of interferon (IFNR), inducing interferon-stimulated gene (ISG) production and activating the adaptive immune response [6]. In this process, LGP2 has been characterized as a balancer of RLR signaling exhibiting either negative or positive regulation of RIG-I- and MDA5-mediated antiviral signaling [7]. In addition, an increasing number of the DEAD-box subfamily from helicases SF2 have been found to have antiviral functions. DDX1 recognizes viral RNA through its helicase domain and activates IFN-I signaling in dendritic cells [8]. DDX6 functions as a co-sensor with RIG-I to bind influenza B virus (IV) RNA and enhance IFN production [9], while also stabilizes hepatitis C virus (HCV) RNA [10]. Moreover, DDX60 is involved in antiviral immunity through both RIG-I-dependent type I IFN production and RIG-I-independent viral RNA decay pathways [11]. Conversely, DDX5 directly interacts with TBK1 and promotes its autophagy-mediated degradation, thereby inhibiting antiviral innate immunity [12].

Helicase superfamily 1 (SF1) and helicases SF2 share a catalytic core with high structural similarity to unwind duplexes [13]. However, SF1 family members rarely participate in host antiviral response. MOV10 restricts viral replication by sequestering vRNPs in the cytoplasm [14] and induces IFN expression via IKKε in a RIG-I–MAVS-independent manner to activate antiviral immunity [15]. ZNFX1 has evolved to function as a dsRNA sensor in mammals, interacting with MAVS to initiate innate antiviral immune responses [16]. On the other hand, highly virulent viral infections or excessive viral loads may lead to hyperactive or prolonged inflammatory responses, potentially triggering cytokine storms that can cause severe tissue damage or multi-organ dysfunction [17]. Consequently, proper and precise modulation of virus-induced inflammatory responses is also essential for maintaining normal cellular homeostasis. Mammalian ZNFX1 can interact with NLRP3 to block it in the cytoplasm, thus inhibiting the inflammatory response [18] and regulate the degradation of host ISGs mRNA by post-transcriptional modification to avoid immune storms [19]. In addition, the remarkable over-expression of ZNFX1 in bivalve and gastropod mollusks infected with dsDNA viruses [20] and brain tissue of asymptomatic Atlantic cod infected with Norodavirus [21] were observed. However, the role of ZNFX1 in the interaction between host cells and aquatic viruses has not been reported.

Although MDA5 and LGP2 in fish and humans are highly conserved in evolution, recent analyses have demonstrated the absence of RIG-I homologous genes in multiple Actinopterygii teleost species, including *Takifugu rubripes*, *Tetraodon nigroviridis, Oryzias latipes*, *Sparus aurata, Dicentrarchus labrax, Siniperca chuatsi* [22–25], *Epinephelus coioides* and *Epinephelus lanceolatus*, which may significantly impair the antiviral defense mechanisms. Therefore, identifying novel nucleic acid receptors and elucidating their immune regulatory mechanisms holds significant scientific value for the development of strategies to control aquatic RNA viral diseases.

Viral nervous necrosis (VNN) is a highly fatal disease of viral aetiology, causing high mortality rates up to 100% in affected larvae and juvenile fish [26]. The impact of VNN extends to over 120 species, encompassing both marine and freshwater fish. Nervous necrosis virus (NNV) is etiological agent of VNN, belonging to the genus *Betanodavirus* of the family Nodaviridae [27]. NNV is an icosahedral, non-enveloped virus containing a genome composed of two positive-sense single-stranded RNAs. RNA1 encodes RNA-dependent RNA polymerase [RdRp, aka Protein A (ProA)] and generates subgenome RNA3 which contains two open reading frames (ORFs) encoding non-structural proteins B1 and B2 [28,29]. RNA2 encodes the capsid protein (CP), the sole structural protein of NNV [30].

In this study, our findings elucidated the antiviral mechanisms of the novel dsRNA receptor ZNFX1 in *E. coioides* (EcZNFX1) lacking RIG-I. EcZNFX1 is a key host protein that is activated during the early stages of OGNNV or *Micropterus salmoides* rhabdovirus (MSRV) infection. We demonstrated that EcZNFX1 bound to OGNNV-derived dsRNA, thereby exerting protective effects to inhibit viral replication on various cell types through balancing inflammatory signals or via a TBK1-dependent innate immune signaling pathway. Our results provide novel insights into a potential antiviral strategy of ZNFX1 in RIG-I-lost-Actinopterygii teleost.

## Results

### EcZNFX1 is relatively primitive

With a long-standing interest in the dynamics of NNV infection in groupers, we identified a notable gap in their innate immune repertoire: species such as *E. coioides* (GenBank: GCA_051314025.1) and *E. lanceolatus* (GCA_005281545.1) conspicuously lack RIG-I, a fundamental dsRNA-sensing receptor vital for antiviral innate immunity. Supporting our focus on compensatory mechanisms, our previous transcriptomic analysis of OGNNV-infected GB cells demonstrated a significant upregulation of the RNA sensor EcZNFX1. Given that mammalian ZNFX1 has been identified as a novel nucleic acid receptor, we hypothesized that EcZNFX1 may function as a compensatory molecule, mitigating the absence of RIG-I in the grouper antiviral response.

**Fig 1:**
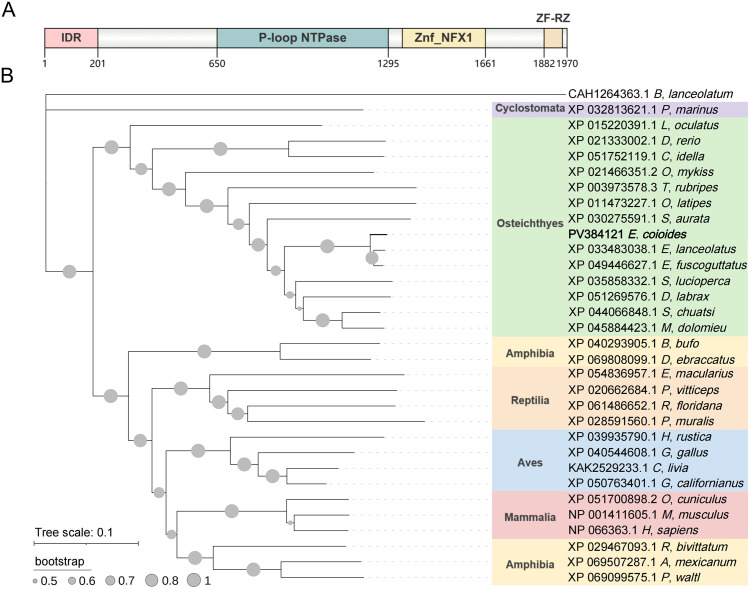
Sequence characterization and phylogenetic analysis. (A) Domains of EcZNFX1. (B) Bayesian phylogenetic tree of 32 ZNFX1 sequences was inferred using the neighbor-joining method in MEGA7, and 10,000 bootstrap replicates.

The open reading frame of EcZNFX1 has been cloned from GB cells and deposited in GenBank under the accession number PV384121. This 5913-bp sequence encodes a 1970-AA protein predicted to contain an N-terminus intrinsically disordered region, a conserved P-loop NTPase, six NFX-1 type zinc fingers, and a C-terminus RZ-type zinc finger domain (Fig 1A). Comparative sequence analysis revealed high conservation of EcZNFX1 among selected teleost species and *Sander lucioperca* (78% identity). In contrast, EcZNFX1 shares lower similarity (51% identity) with its *Homo sapiens* homolog and notably lacks the ARM domain (S1 Fig). Phylogenetic analysis demonstrated that teleost ZNFX1, especially grouper ZNFX1, are relatively primitive in evolutionary status and have a significant genetic distance from the ZNFX1 of higher vertebrates (mammals, aves, reptilia and amphibian) (Fig 1B).

### EcZNFX1 functions as an ISG responsive to RNA virus infection

**Fig 2:**
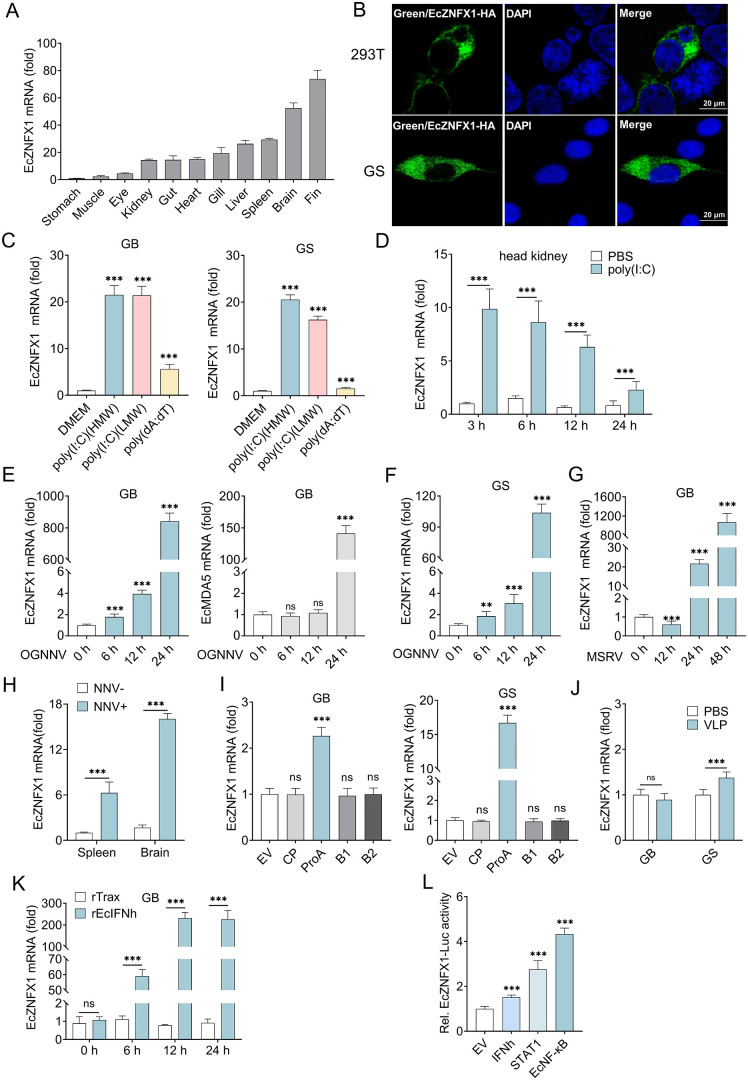
OGNNV infection upregulates the expression of EcZNFX1. (A) Tissue distribution and relative transcription (mRNA levels) of EcZNFX1 was analyzed by qRT-PCR. (B) The subcellular localization of EcZNFX1 in GS and HEK-293Tcells. The experiments were repeated three times, independently, with similar results. (C) GB and GS cells were stimulated with poly(I:C)(HMW), poly(I:C)(LMW) and poly(dA:dT) for 6 h. The mRNA levels of EcZNFX1 were analysed by qRT-PCR. (D) The mRNA level of EcZNFX1 in the head kidney of orange-spotted grouper stimulated by poly(I:C) was detected by qRT-PCR analysis. (E-G) The mRNA levels of EcZNFX1 or EcMDA5 in OGNNV-infected GB cells, OGNNV-infected GS cells, and MSRV-infected GB cells were detected by qRT-PCR analysis. (H) The mRNA levels of EcZNFX1 in brain and spleen of healthy or OGNNV-infected orange-spotted grouper were detected by qRT-PCR analysis. (I and J) GB and GS cells were transiently transfected with the indicated expressing plasmids for 24 h or stimulated with VLP (1 µg/mL) for 24 h. The mRNA levels of EcZNFX1 were analysed by qRT-PCR. (K) EcZNFX1 expression was measured by qRT-PCR in GB cells were treated with 1 μg/mL rEcIFNh for the indicated time. (L) Luciferase activity analysis of *EcZNFX1* promoter in GS cells transfected with the indicated expressing plasmids.The data are shown as the means ± SDs. Statistical differences were determined by one-way ANOVA (C, E, F, G, I and L) and Student t-test (D, H, J and K). *P < 0.05, **P < 0.01, ***P < 0.001, ns, no significance.

Tissue-specific distribution profiling showed that EcZNFX1 was predominantly expressed in fin, brain, and spleen (Fig 2A), with GB and GS cells originating from the latter two highly expressing organs. It was exclusively localized in the cytoplasm (Fig 2B) at the subcellular level. To confirm whether EcZNFX1 was involved in response to viral infection, its expression pattern was detected in both GB and GS cells treated with different stimuli, including nucleic acid analogues stimulation, virus infection, viral protein ectopic expression, VLP invasion, and IFN-I incubation. EcZNFX1 was significantly upregulated, whether in response to nucleic acid analogs stimulation in cultured cells (Fig 2C) or following poly(I:C) injection in grouper (Fig 2D). Notably, although EcIFIT1, as an ISG, was significantly upregulated under both RNA and DNA analog stimulation, RNA analogs poly(I:C)(HMW) induced much stronger EcZNFX1 activation compared to the DNA analog poly(dA:dT) (S2A Fig).

RNA virus infection also activates EcZNFX1. After 24 h of OGNNV and MSRV infection, the expression level of EcZNFX1 significantly increased (Fig 2E-2G). Interestingly, EcZNFX1 expression in GB cells was activated as early as 6 h post OGNNV infection, which is much earlier and also stronger than that of EcMDA5 (Fig 2E). Similarly, in the tissues of OGNNV-infected grouper, EcZNFX1 expression was increased, with a significantly higher increase observed in brain compared to spleen (Fig 2H). Furthermore, only the ectopic expression of ProA among the four NNV-encoded proteins was able to significantly augment EcZNFX1 (Fig 2I), which should be related to the ability of ProA to generate dsRNA intermediates [31]. Since VLPs, serving as mimics of NNV, possess the capability for viral entry without the ability to proliferate, we utilized VLPs in the invasion experiments. After VLP invasion, a slight increase in EcZNFX1 transcripts was observed in GS cells, suggesting that EcZNFX1 is essentially not involved in NNV entry (Fig 2J) as well as the heterogeneity in the response to OGNNV infection between GS and GB cells. Interestingly, after infection with OGNNV at the same MOI, the proliferation of OGNNV in GB cells is significantly faster than that in GS cells (S2B Fig). Similarly, the viral load of OGNNV in the brain tissue of OGNNV-infected groupers is also significantly higher than that in the spleen (S2C Fig). This observation reflects the differences in the antiviral capacity of different cells and tissues against OGNNV.

We next examined whether ZNFX1 is an ISG by incubating GB cells with recombinant EcIFNh. The results of qRT–PCR showed that the expression of EcZNFX1 could be induced by EcIFNh (Fig 2K). The bioinformatics analysis of its promoter region revealed that the promoter of Ec*Znfx1* contains the binding motifs of two transcription factor, EcNF-κB and EcSTAT1, in a relatively concentrated and overlapped region. Subsequent reporter assays showed that the expression of reporter gene could be induced by the Ec*Znfx1* promoter in the presence of EcNF-κB and EcSTAT1 (Fig 2L). All these data indicate EcZNFX1 functions as an ISG and can respond to RNA virus infection, potentially being involved in the intracellular host-virus interaction process.

### EcZNFX1 inhibits the replication of OGNNV

**Fig 3:**
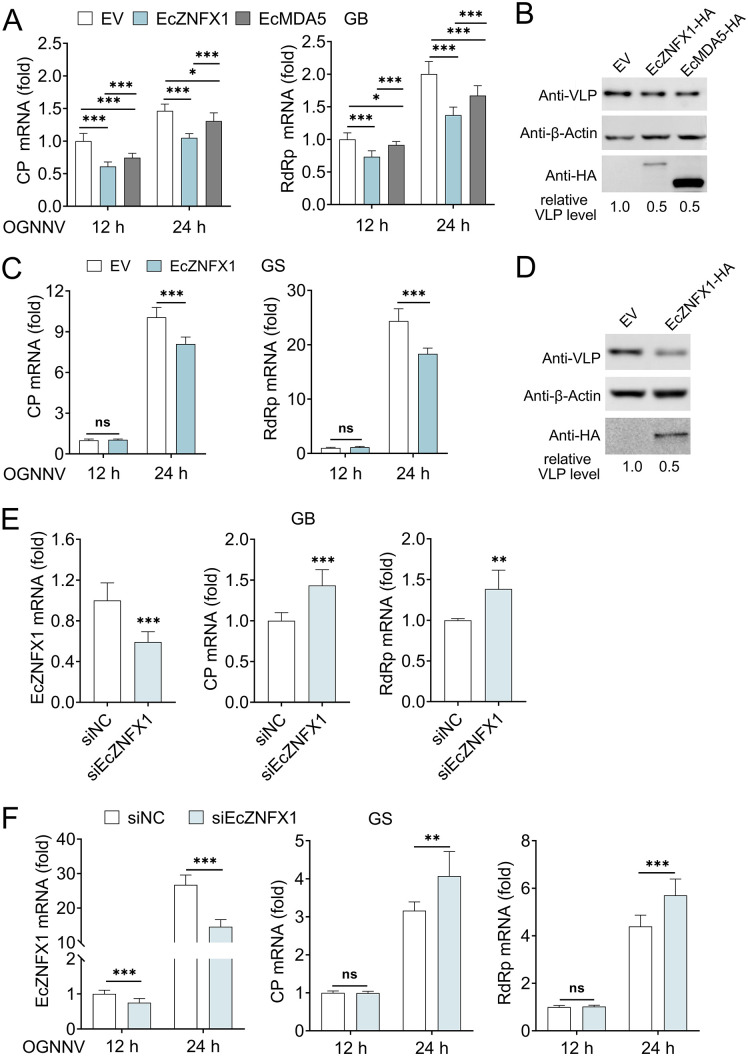
EcZNFX1 inhibits the replication of OGNNV in vitro. (A and C) The mRNA level of OGNNV CP and RdRp in OGNNV-infected GB and GS cells transfected with the indicated expressing plasmids was measured by qRT-PCR. (B and D) The protein expression of OGNNV CP was evaluated by Western blotting in OGNNV-infected GB and GS cells transfected with the indicated plasmids. (E and F) The mRNA levels of EcZNFX1, OGNNV CP and RdRp in OGNNV-infected GB and GS cells transfected with siRNAs targeting control (siNC) or EcZNFX1 (siEcZNFX1) were detected by qRT-PCR. The data are shown as the means ± SDs. Statistical differences were determined by two-way ANOVA (A) and Student t-test (C, E and F). *P < 0.05, **P < 0.01, ***P < 0.001, ns, no significance.

To clarify the effects of EcZNFX1 on OGNNV infection, EcZNFX1 overexpression or siRNA-mediated knockdown were performed in GB and GS cells before OGNNV infection. Overexpression of EcZNFX1 significantly inhibited (Fig 3A-3D), while knockdown of EcZNFX1 promoted (Fig 3E and 3F) OGNNV replication, as evidenced by alterations in the mRNA levels of CP and RdRp, and protein level of CP. Importantly, the inhibitory effect of EcZNFX1 on OGNNV replication in GB cells was more pronounced compared to that of EcMDA5 (Fig 3A). Collectively, compared to EcMDA5, EcZNFX1 responds earlier to OGNNV infection and exhibits stronger antiviral activity, indicating a more pivotal role in the grouper’s defense against OGNNV.

**Fig 4:**
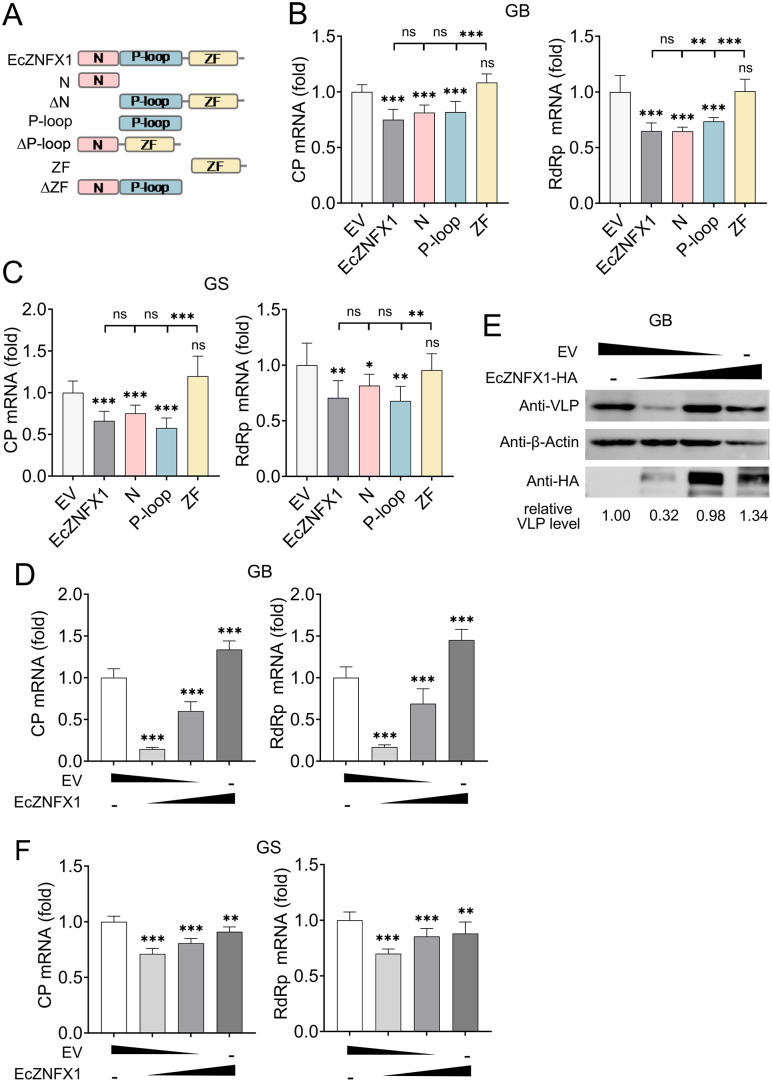
Identification of the key structural domain and dose-dependent antiviral activity of EcZNFX1. (A) Schematic diagram of EcZNFX1 and its domain-deleted mutants. (B and C) The mRNA level of OGNNV CP and RdRp in OGNNV-infected GB and GS cells transfected with the indicated expressing plasmids was examined by qRT-PCR analysis. (D and F) The mRNA level of OGNNV CP and RdRp in OGNNV-infected GB and GS cells transfected with different doses of EcZNFX1 expressing plasmids was examined by qRT-PCR analysis. (E) The protein expression of OGNNV CP was evaluated by Western blotting in OGNNV-infected GB cells transfected with different doses of indicated expressing plasmids. The data are shown as the means ± SDs. Statistical differences were determined by one-way ANOVA. *P < 0.05, **P < 0.01, ***P < 0.001, ns, no significance.

To identify the key domain of EcZNFX1 responsible for its antiviral activity, we constructed and overexpressed truncated mutants prior to OGNNV infection. Similar to samples containing the wild-type EcZNFX1, the mRNA levels of OGNNV were still inhibited in samples containing the N-terminal domain and P-loop NTPase domain, but no reduction was observed in samples with the zinc finger domain (Fig 4B and 4C). Furthermore, the excessive EcZNFX1 impaired its antiviral function (Fig 4D-4F), suggesting that EcZNFX1 must be maintained at a certain level to effectively exert its antiviral capability. These findings demonstrate that EcZNFX1 exerts its antiviral properties via the N-terminal domain and P-loop NTPase domain in a dose-independent manner.

### EcZNFX1 directly binds to OGNNV dsRNA intermediates via its P-loop NTPase domain

**Fig 5:**
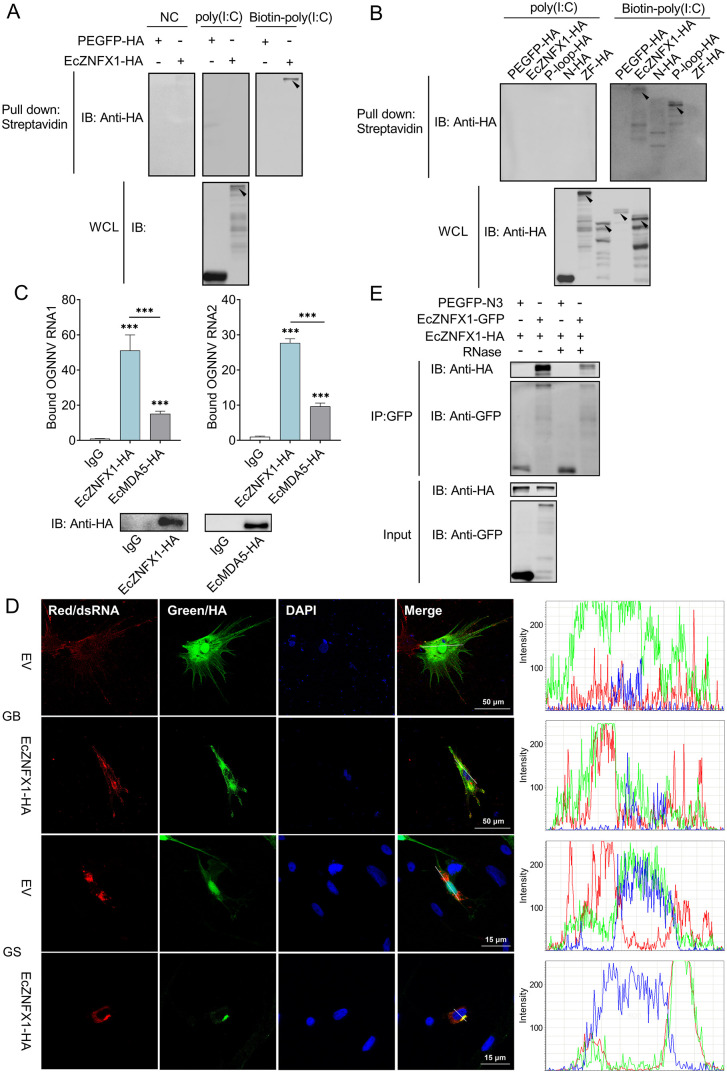
EcZNFX1 directly binds to ORNNV dsRNA through the P-loop NTPase domain. (A and B) HEK-293T cells were transfected with indicated expression vectors. Cell lysate was incubated with Biotin-poly(I:C)(HMW) or non-conjugated poly(I:C). Biotin-poly(I:C)(HMW) was pull-downed with streptavidin beads. Samples were subjected to SDS-PAGE, and proteins were detected by Western blotting. (C) GB cells were transfected with the indicated plasmids, infected with OGNNV, subjected to RNA immunoprecipitation (RIP) and analysed by qRT-PCR to quantify the bound OGNNV-RNA. (D) GB and GS cells were transfected with EcZNFX1 expressing plasmids and infected with OGNNV for 24 h, then fixed and stained for EcZNFX1-HA, dsRNA and DAPI. The experiments were repeated three times, independently, with similar results. (E) Co-immunoprecipitation and Western blot analysis of HEK-293T cells transfected with EcZNFX1-flag and EcZNFX1-HA. The data are shown as the means ± SDs. Statistical differences were determined by one-way ANOVA (C). *P < 0.05, **P < 0.01, ***P < 0.001, ns, no significance.

Mammalian ZNFX1 has been verified to directly bind to cytoplasmic RNA [16,32]. Based on the above findings, we hypothesize that EcZNFX1 may act as a dsRNA sensor to compensate for the absence of RIG-I in orange-spotted grouper. To confirm whether EcZNFX1 can bind to dsRNA, in vitro RNA pulldown assay was performed. Eukaryotic expressed HA-tagged EcZNFX1 could only be detected in Biotin-poly(I:C) sample which was precipitated by streptavidin beads (Fig 5A), proving that EcZNFX1 binds to poly(I:C) directly. To further assess the specific domain of EcZNFX1 responsible for the binding, three truncated mutants of EcZNFX1 were utilized in pulldown assay and only the mutant with P-loop NTPase domain could be detected (Fig 5B). To verify EcZNFX1’s recognition ability to viral RNA, RIP assays were carried out after OGNNV infection of EcZNFX1- or EcMDA5-overexpressing GB cells. The direct binding of EcZNFX1 to viral RNA1 and RNA2 were confirmed by RT-qPCR, with a significantly higher binding affinity compared to those of EcMDA5 (Fig 5C). Co-localization of EcZNFX1 with viral dsRNA intermediates was observed in EcZNFX1-overexpressed and OGNNV-infected GB and GS cells, indicating direct recognition of OGNNV-derived dsRNA by EcZNFX1 (Fig 5D). Furthermore, Co-IP assay with the addition of RNase revealed that EcZNFX1 can undergo oligomerization independently of RNA bridging (Fig 5E), which is a characteristic of RNA sensor. These results suggest that EcZNFX1 directly and specifically interacts with OGNNV dsRNA intermediates via its P-loop NTPase domain.

### ZNFX1 regulates innate immune balance in GB cells

**Fig 6:**
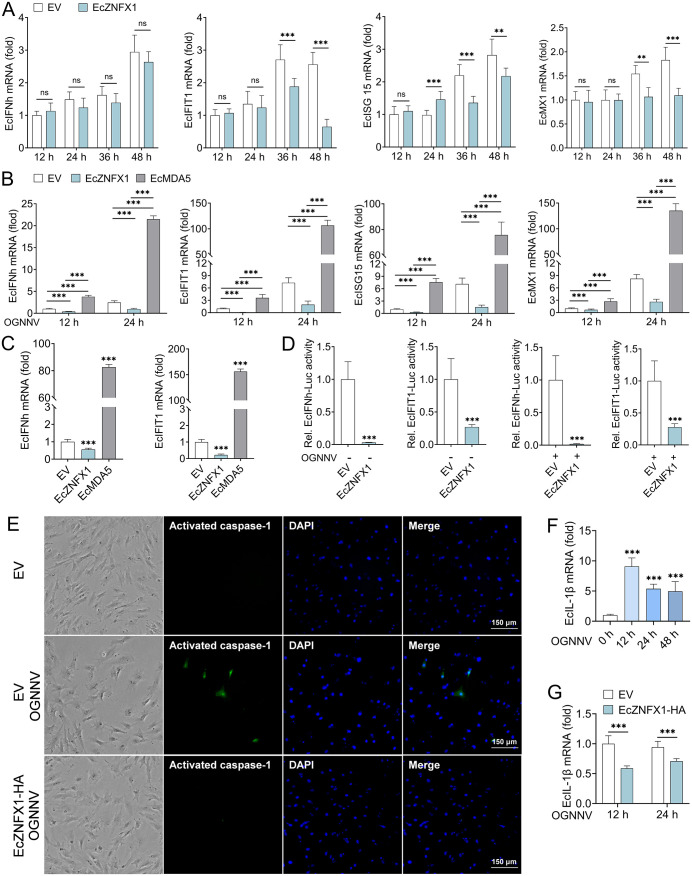
EcZNFX1 regulates innate immune balance in GB cells. (A and B) The mRNA transcripts of indicated IFN and ISGs were analysed by qRT-PCR in GB cells or OGNNV infected GB cells transfected with indicated expression vectors. (C) EcIFNh and EcIFIT1 mRNA transcripts were analysed by qRT-PCR in GB cells transfected with indicated expression vectors followed by poly(I:C) stimulation for 6 h. (D) Luciferase activity analysis of EcIFNh and EcIFIT1 promoters in GB cells or OGNNV-infected GB cells transfected with the indicated expressing plasmids. (E) GB cells were transfected with indicated expression vectors for 12 h, and challenged with OGNNV for 36 h. Intracellular activated caspase-1 was stained with FAM-FLICA reagent. (F) The mRNA levels of EcIL-1β in OGNNV-infected GB cells were detected by qRT-PCR analysis. (G) The mRNA level of EcIL-1β in OGNNV-infected GB cells transfected with the indicated expressing plasmids was measured by qRT-PCR. The data are shown as the means ± SDs. Statistical differences were determined by Student t-test (A and, D and G) and one-way ANOVA (B, C and F). *P < 0.05, **P < 0.01, ***P < 0.001, ns, no significance.

Classic cytosolic dsRNA receptors, such as RIG-I and MDA5, are well-characterized inducers of IFN production via RLR signaling upon viral nucleic acid detection. To determine whether EcZNFX1 exerts antiviral effects through this pathway in GB cells, we overexpressed EcZNFX1 and analyzed the transcriptional levels and promoter activities of IFN-I and ISGs. Unexpectedly, EcZNFX1 overexpression significantly attenuated the mRNA levels of key ISGs (Fig 6A), including EcIFIT1, EcISG15, and EcMX1, in GB cells. Furthermore, EcZNFX1 overexpression suppressed the mRNA levels of EcIFNh, EcIFIT1, EcISG15, and EcMX1 triggered by OGNNV infection (Fig 6B). Similarly, in GB cells stimulated with poly(I:C), EcZNFX1 overexpression reduced the expression of EcIFNh and EcIFIT1 (Fig 6C). Notably, these inhibitory effects were not observed in GB cells overexpressing EcMDA5 (Fig 6B and 6C). In addition, EcZNFX1 significantly suppressed the promoter activities of both EcIFNh and EcIFIT1 (Fig 6D). Remarkably, EcZNFX1 also inhibited pyroptosis induced by OGNNV infection, as evidenced by the weakened caspase-1 activity and suppressed EcIL-1β expression triggered by OGNNV infection (Fig 6E-6G). Given that human ZNFX1 has been confirmed to modulate IFN balance during viral infection to avoid excessive immune response [19], we speculate that in GB cells, the nerve cells with limited regenerative capacity, EcZNFX1 may independently inhibit OGNNV proliferation outside of IFN-related signaling pathways. This suppression of inflammation could represent a protective mechanism to limit cellular damage during viral challenge in brain.

### EcZNFX1 interacts with EcTBK1 to initiate IFN response in GS cells

**Fig 7:**
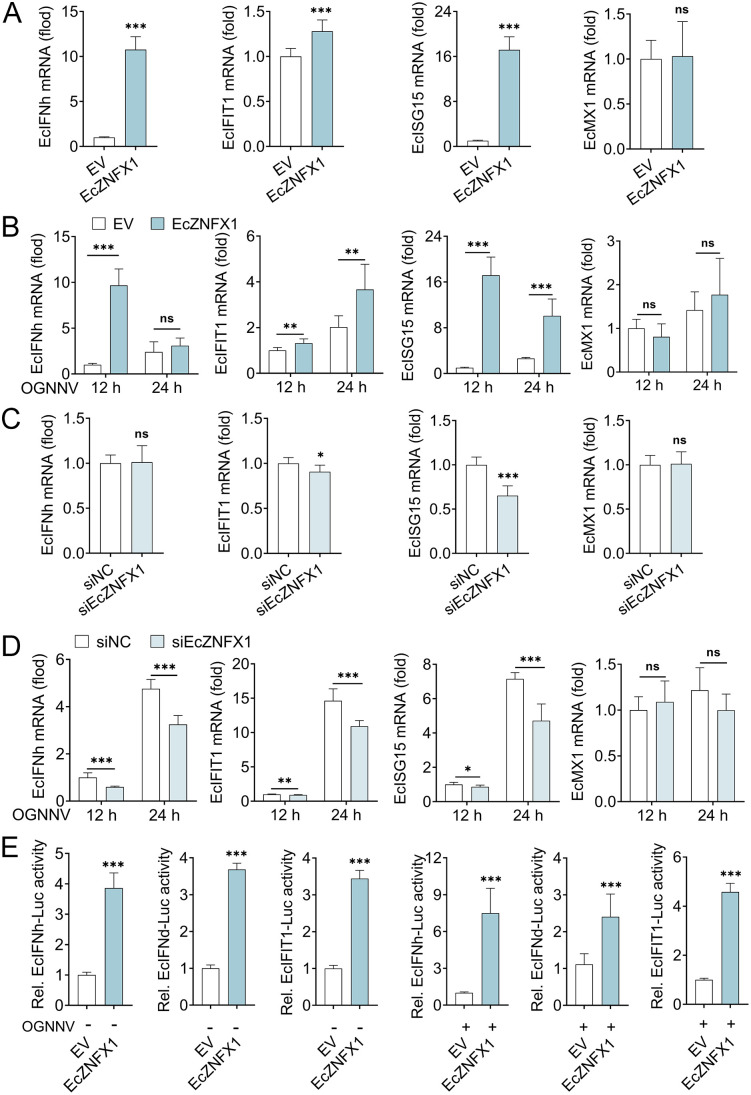
EcZNFX1 initiates IFN response in GS cells. (A and B) The mRNA levels of indicated IFN and ISGs were analysed by qRT-PCR in GS cells or OGNNV infected GS cells transfected with indicated expression vectors. (C and D) The mRNA levels of indicated IFN and ISGs in GS cells or OGNNV infected GS cells transfected with siRNAs against control (siNC) or EcZNFX1 (siEcZNFX1) were assessed by qRT-PCR. (E) Promoter activity of *EcIFNh, EcIFNd and EcIFIT1* in GS cells or OGNNV infected GS cells transfected with the indicated expressing plasmids was detected by Luciferase activity analysis. The data are shown as the means ± SDs. Statistical differences were determined by Student t-test. *P < 0.05, **P < 0.01, ***P < 0.001, ns, no significance.

The teleost spleen exhibits more robust innate and adaptive immune activities compared to the brain, suggesting its importance in systemic antiviral defense. Given the previous finding that GB and GS cells exhibit distinct differences in antiviral capacity during OGNNV infection, we further investigated how EcZNFX1 regulates the IFN pathway in GS cells. EcZNFX1 overexpression markedly increased the mRNA transcripts of EcIFNh, EcIFIT1, and EcISG5, regardless of OGNNV infection (Fig 7A and 7B). Conversely, EcZNFX1 knockdown significantly attenuated their expression (Fig 7C and 7D). Furthermore, the promoter activity of EcIFNh, EcIFNd, and EcIFIT1 in GS cells was significantly enhanced by EcZNFX1 overexpression (Fig 7E). These findings demonstrate that EcZNFX1 functions as a positive regulator of IFN response in spleen cells, reinforcing its role in cell-type-specific antiviral immunity.

**Fig 8:**
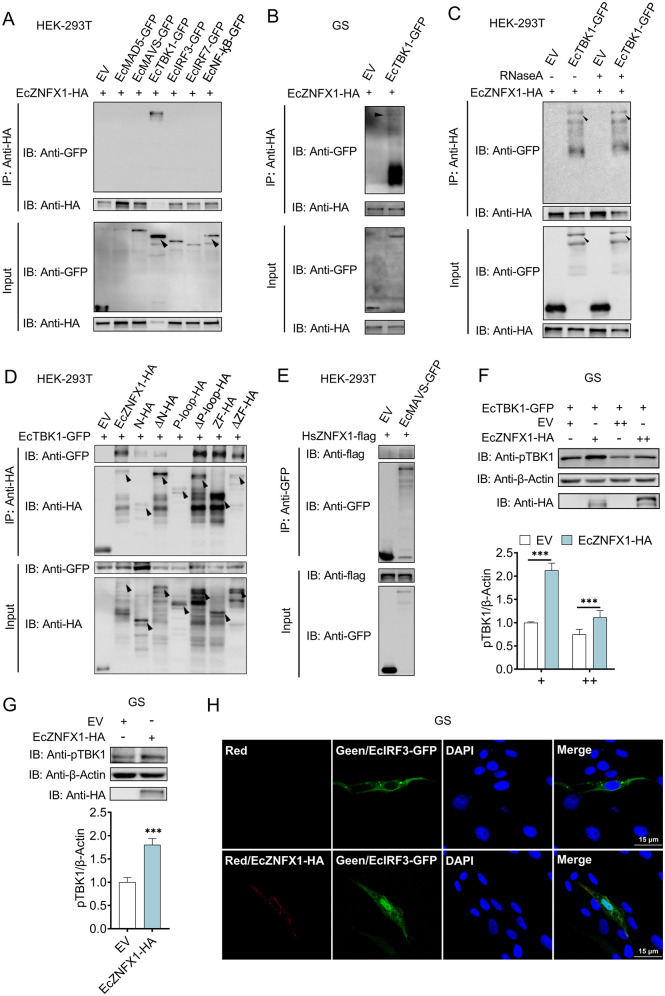
EcZNFX1 interacts with EcTBK1 and regulates its activity. (A) Co-IP analysis of HEK-293T cells transfected with GFP-tagged empty vector, EcMDA5, EcMAVS, EcTBK1, EcIRF3, EcIRF7 and EcNF-κB, together with EcZNFX1-HA. (B) Co-IP analysis of GS cells transfected with GFP-tagged empty vector and EcTBK1, together with EcZNFX1-HA. (C) Co-IP analysis of EcTBK1 and EcZNFX1 in the presence of RANase. (D) Co-IP analysis of HEK-293T cells transfected with EcTBK1-GFP and domain-deleted mutants of EcZNFX1 or empty vector. (E) Co-IP analysis of HEK-293T cells transfected with GFP-tagged empty vector and EcMAVS, together with HsZNFX1-flag. (F and G) Western blot analysis of the endogenous and exogenous phosphorylated EcTBK1 in EcZNFX1 overexpressed GS cells. (H) GS cells were transfected with EcIRF3-GFP and EcZNFX1-HA or empty vector for 36 h. The nuclear translocation of EcIRF3 was detected by Immunofluorescence. The experiments were repeated three times, independently, with similar results. The data are shown as the means ± SDs. Statistical differences were determined by Student t-test. *P < 0.05, **P < 0.01, ***P < 0.001, ns, no significance.

In humans, ZNFX1 initiates IFN signaling by interacting with MAVS via its ARM domain. However, teleost ZNFX1 orthologs, including EcZNFX1, lack a conserved ARM domain. To clarify the EcZNFX1-interacting effectors in GS cells, we systematically assessed protein-protein interactions within the basic components of RLR pathway. Leveraging the benefits of high transfection efficiency and exclusion of potential interference from endogenous fish proteins, Co-IP assays performed in HEK 293T cells confirmed a specific interaction between EcZNFX1 and EcTBK1, with no detectable binding to EcMAVS (Fig 8A). This distinctive finding was subsequently validated in GS cells, a native fish cell context (Fig 8B). Notably, this interaction remained intact even in the presence of RNase (Fig 8C), effectively ruling out RNA-mediated bridging effect.

Domain deletion mapping (schematic in Fig 4A) identified that both the N-terminal domain and the zinc finger domain play crucial roles in mediating the EcZNFX1-EcTBK1 interaction (Fig 8D). Furthermore, we found that human ZNFX1 can interact with fish EcMAVS (Fig 8E). Given that human ZNFX1 contains an ARM domain absent in its teleost ortholog, EcZNFX1 (S1 Fig), this structural differences provide a plausible explanation for why EcZNFX1 selectively interacts with EcTBK1 rather than EcMAVS.

Besides confirming the interaction, functional validation demonstrated that EcZNFX1 overexpression enhanced the phosphorylation of EcTBK1 (pTBK1) in GS cells, whether EcTBK1 was exogenous (Fig 8F) or endogenous (Fig 8G). After EcTBK1 activation, its downstream EcIRF3 was revealed to be efficiently translocated to the nuclear by immunofluorescence analyses (Fig 8H). Collectively, our findings establish a ZNFX1-TBK1-IRF3 signaling axis in GS cells. Upon viral dsRNA recognition, EcZNFX1 interacts with EcTBK1 through its N-terminal domain and the zinc finger domain, and activates TBK1, subsequently initiating the IFN-I immune response.

### EcZNFX1 functionally compensates for RIG-I

**Fig 9:**
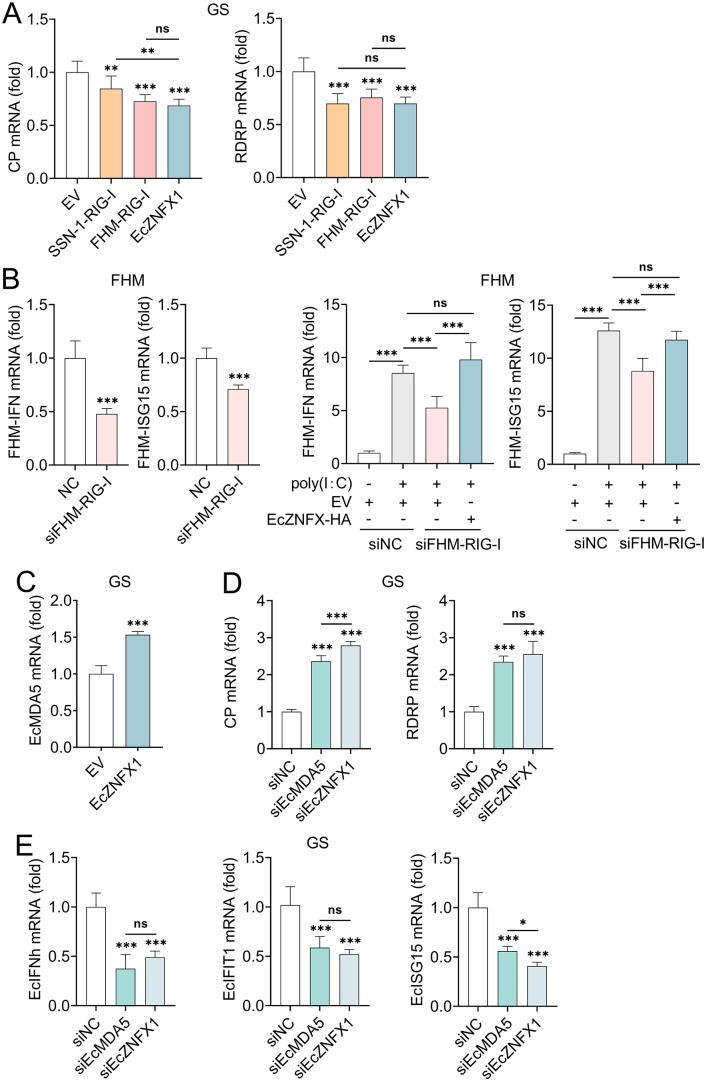
EcZNFX1 functionally compensates for RIG-I. (A) The mRNA level of OGNNV CP and RdRp in OGNNV-infected GS cells transfected with the indicated expressing plasmids was measured by qRT-PCR. (B) The mRNA levels of FHM-IFN and FHM-ISG15 in FHM cells transfected with siRNAs targeting control (siNC) or FHM-RIG-I (siFHM-RIG-I) with or without poly(I:C) stimulation were detected by qRT-PCR. (C) EcMDA5 mRNA transcripts were analysed by qRT-PCR in GS cells transfected with EcZNFX1 expression vectors and infected with OGNNV. (D and E) CP, RDRP, EcIFNh, EcIFIT1 and EcISG15 mRNA levels in GS cells transfected with siRNAs targeting control (siNC), EcMDA5 (siEcMDA5) or EcZNFX1 (siZNFX1) and infected with OGNNV were assessed by qRT-PCR. The data are shown as the means ± SDs. Statistical differences were determined by one-way ANOVA (A, B, D and E) andStudent t-test (B and C). *P < 0.05, **P < 0.01, ***P < 0.001, ns, no significance.

The absence of the canonical RNA sensor RIG-I in Actinopterygii species creates a critical knowledge gap concerning their early detection of viral RNA to establish the antiviral immune response. EcZNFX1 has been shown to initiate IFN signaling through interacting with EcTBK1, positioning this protein as a prime candidate to fill this void. To test this hypothesis, the antiviral capacities of RIG-I of fathead minnow (FHM) and striped snakehead (SSN-1) were assessed in GS cells. Remarkably, FHM-RIG-I/SSN-1-RIG-I overexpression inhibited OGNNV proliferation with an efficiency comparable to that of EcZNFX1 (Fig 9A). Moreover, in RIG-I knocked down FHM cells, EcZNFX1 overexpression successfully restored the activation of IFN and ISGs (Fig 9B), provding strong functional evidence that ZNFX1 can compensate for RIG-I deficiency.

Since groupers retain EcMDA5, another well-established key dsRNA sensor, we aimed to clarify the relationship and specific contribution differences between EcZNFX1 and EcMDA5 in antiviral responses. The previous results in GB cells showed that, compared with EcMDA5, EcZNFX1 exhibits a more robust and rapid response to OGNNV infection (Fig 2E). Nevertheless, their antiviral mechanisms diverge: MDA5 activates the IFN response via the canonical RLR pathway, whereas ZNFX1 suppresses it. In contrast, both proteins activate the IFN response against OGNNV within GS cells. EcZNFX1 overexpression further activate the expression of EcMDA5 (Fig 9C). Additionally, we performed a comparative knockdown assay in GS cells. Althought silencing either EcZNFX1 or EcMDA5 downregulated the expression of IFN and ISGs and increased OGNNV loads (Fig 9D and 9E), varying knockdown efficiencies between the two genes prevented a definitive comparison of their relative contributions to the antiviral process.

Collectively, these findings support a model where EcZNFX1 acts as an early dsRNA sensor in groupers. It can be rapidly expressed in the early stage of viral infection and functionally compensate for the absence of RIG-I, thereby helpping to establish a more robust antiviral defense.

## Discussion

Owing to the imperfect development of adaptive immunity, teleost, especially the juvenile and larval primarily rely on innate immunity to resist pathogen invasions. During RNA virus invasion, RLRs, such as RIG-I and MDA5, recognize viral nucleic acids and activate downstream interferon signaling [33]. Although MDA5 is highly conserved across fish and higher vertebrates, orthologues of RIG-I have not been identified in many Actinopterygii teleosts [22–25] including *E. coioides*. In the event of gene inactivation or loss, organisms can maintain homeostasis via a homology-dependent genetic compensation response (HDGCR). Under the pressure of viral infection, the absence of RIG-I may be compensated by the functional replacement mechanisms. Therefore, identifying potential novel nucleic acid receptors in Actinopterygii that could complement the antiviral function of RIG-I is crucial for developing innovative strategies for RNA virus control. Mammalian ZNFX1 is recognized as a novel cytoplasmic nucleic acid receptor to initiate the innate immune response [16]. However, whether ZNFX1 in lower vertebrates can function in viral infection remains unclear.

Here, we found Actinopterygii ZNFX1 is evolutionary primitive and exhibits a significant genetic distance from higher vertebrates. As an IFN-stimulated gene, EcZNFX1 can recognize viral dsRNA and exert antiviral effects. EcZNFX1 expression was significantly upregulated after stimulation with poly(I:C) or poly(dA:dT), or infection with either the single-stranded negative-sense RNA virus OGNNV or the single-stranded positive-sense RNA virus MSRV. Notably, compared with EcMDA5, EcZNFX1 exhibits a faster response speed and more potent inhibitory effect on OGNNV proliferation. Moreover, ZNFX1 is capable of further inducing MDA5 expression, thereby contributing to the establishment of the antiviral response. As the first line of defense against pathogen invasion, innate immunity is characterized by rapid response and broad-spectrum defense, which can effectively limit pathogen spread and provide a critical window for the development of adaptive immunity [34]. Emerging evidence indicates that certain PRRs, like TLR5 [35] and cGAS [36] of Actinopterygii, can recognize multiple PAMPs, such as DNA and RNA. Given these characteristics, we propose that the evolutionarily conserved ZNFX1 may facilitates the rapid establishment of a broad-spectrum and effective antiviral response in Actinopterygii teleosts, which possess relatively primitive immune systems.

As a member of the RNA helicase SF1 family, mammalian ZNFX1 has been demonstrated to bind both viral-derived double-stranded RNA (dsRNA) and cellular mRNA [16,32]. EcZNFX1 possesses a conserved P-loop NTPase domain with nucleic acid binding sites on its surface, suggesting its potential to bind dsRNA [37]. Our results demonstrated that EcZNFX1 directly recognizes OGNNV-derived dsRNA, achieved through its P-loop NTPase domain, and exhibits a stronger binding affinity compared to EcMDA5. However, the binding affinity and specificity of EcZNFX1 for different nucleic acid types, as well as its mechanisms in regulating target RNA metabolism, require further elucidation.

In terms of specific action mechanism, EcZNFX1 exhibits distinct characteristics compared to MDA5. Upon detection of exogenous dsRNA, MDA5 undergoes dephosphorylation and ubiquitination of its N-terminal CARD domain, which subsequently binds to MAVS [38]. While human ZNFX1 interacts with MAVS via its N-terminal ARM domain [16], the absence of the ARM domain suggests EcZNFX1 may regulate innate immune through alternative routes. Previous studies have demonstrated that members of the SF2 family, such as DDX3 [39,40] and DDX5 [12], can interact with TBK1 or IKKε and regulates its activity. We found that EcZNFX1 can act as a complementary receptor to RIG-I, directly interacting with EcTBK1 through its N-terminal and zinc finger domain, and activating MAVS-independent phosphorylation of EcTBK1. This interaction promotes EcIRF3 nuclear translocation and subsequent induction of IFN expression. This MAVS-independent signaling may help cells establish an antiviral response more rapidly.

On the contrary, in GB cells, EcZNFX1 inhibits OGNNV-induced pyroptosis and downregulates the expression of IFN-I and ISGs. Mammalian ZNFX1 suppresses NLRP3 inflammasome activation [18] in lipopolysaccharide stimulated mice and accelerates the decay of IFN and ISGs mRNA during viral infection, thereby protecting cells from immune storms [19]. The intrinsic characteristics and external microenvironment of the central nervous system (CNS) pose significant challenges for tissue repair. Neurons, being highly differentiated cells, display restricted regenerative potential after injury. Additionally, the CNS injury environment, characterized by inhibitory myelin debris and inflammatory factors, further impedes neuronal regeneration [41]. In GB cells with heightened NNV susceptibility and attenuated regenerative capacity, the initiation of an immune storm upon NNV infection may lead to irreversible damage. EcZNFX1 may function as a buffer for inflammatory responses, thereby maintaining the immune privilege of the nervous system and protecting neural tissue from damage caused by excessive inflammatory reactions. Thus, EcZNFX1 has a dual regulatory effect on modulating virus-induced inflammatory responses in different cellular contexts.

Several helicases play pivotal roles in modulating viral genome stability and translation, thereby shaping host-virus interactions. For example, DDX6, a SF2 helicase, improves the stability of HCV RNA to promote viral replication [10]. The SF1 helicases member, MOV10, restricts HIV proliferation by sequestering viral ribonucleoprotein complex (vRNP) in the cytoplasm through interaction with HIV nuclear protein (NP) [14], and directly inhibits HCV replication independently of its helicase or ATPase activity via RNA binding capability [42]. *Caenorhabditis elegans* ZNFX1 binds RNAi-targeted transcripts, sequesters them into perinuclear phase-separated condensates, and promotes small RNA (sRNA) amplification [43]. Mammalian ZNFX1 regulates the half-life of cytoplasmic mRNA [19,32]. Consequently, we propose that EcZNFX1 may inhibit viral replication in GB cells through two potential mechanisms: (i) direct binding to viral RNA to impede virions assembly, and (ii) regulation of target RNA stability to suppress viral replication.

Notably, OGNNV proliferation is significantly more pronounced in orange-spotted groupers brain tissue and GB cells compared to spleen cells and GS cells. This discrepancy may be attributed to the spleen’s robust immune capacity and the cell-type-specific immune regulatory mechanisms by EcZNFX1. For example, in response to OGNNV infection, the pyroptotic signaling pathways in GB and GS cells exhibit opposite responses. The specific mechanism by which EcZNFX1 inhibits OGNNV proliferation in GB cells and its role in post-transcriptional modifications remain to be further explored. It should be noted that an important limitation of this study was the absence of grouper-specific antibodies, which precluded the protein analysis of the grouper signaling pathway, such as EcZNFX1, EcIRF3. Future studies utilizing specific anti-EcZNFX1 antibodies are essential to confirm the observed expression patterns and functional attributes, thereby providing a more comprehensive validation of our findings.

## Conclusions

**Fig 10:**
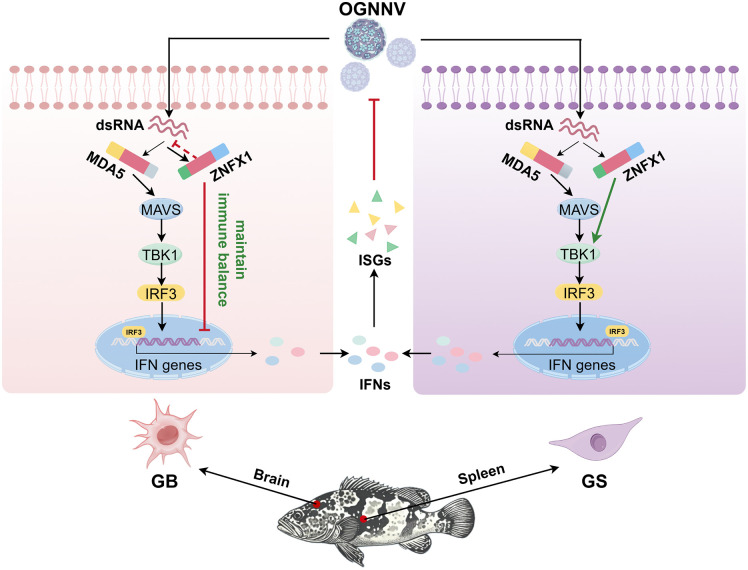
Schematic mechanism of cell-type-specific antiviral effect of EcZNFX1 in orange-spotted grouper lacking RIG-I. In GB cells with high OGNNV susceptibility and attenuated regenerative capacity, EcZNFX1 inhibits IFN-I/ISGs production and also pyroptosis induced by OGNNV infection, thereby avoiding excessive immune response to protect neurons. In GS cells with stronger immune capacity, EcZNFX1 activates the antiviral IFN-I signaling by directly interacting with TBK1. These findings highlight the dual roles of EcZNFX1 in antiviral defense (By Figdraw).

Our study identifies EcZNFX1 as a novel dsRNA receptor that inhibits RNA virus replication during early infection while exhibiting cell-type-specific immunomodulation. In highly susceptible nerve cells with limited regenerative capacity, EcZNFX1 suppresses OGNNV-triggered IFN response and pyroptosis, leading to a less inflammatory but more protective immune reaction. Conversely, in splenocytes, EcZNFX1 activates TBK1-dependent IRF3 pathway to robustly induce the IFN-I immune response, establishing a dual regulatory framework for balancing antiviral defense and immune-mediated pathology. These findings highlight EcZNFX1 as a compensatory receptor in RIG-I-deficient teleosts, illustrating evolutionary adaptations in PRR networks of Actinopterygii fish (Fig 10).

## Materials and methods

### Ethics statement

The study is compliant with all relevant ethical regulations for animal experiments. All the experimental protocols were approved by the Institutional Animal Care and Use Committee of Sun Yat-sen University.

### Fish, cell lines and virus

Healthy orange-spotted grouper individuals (body weight of 10 g) were sourced from Liangshi Aquatic Seed Industry Co., LTD (Guangdong, China) and acclimated for one week in a recirculating seawater aquaculture system. Healthy or OGNNV-infected moribund juvenile orange-spotted groupers were collected in Hainan, China. 

The grouper brain cell (GB) and grouper spleen cell (GS) were established by Mr. Chuanfu Dong and cultured in Dulbecco’s modified Eagle medium (DMEM medium) with 10% fetal bovine serum at 27°C with 5% CO_2_. FHM cells were cultured in M199 medium with 10% (vol/vol) fetal bovine serum (FBS) at 27°C with 5% CO_2_. HEK-293T cells were maintained at 37°C with 5% CO_2_ in DMEM supplemented with 10% FBS. OGNNV-HN1 (GenBank accession numbers MG874757.1 and MG874758.1) was isolated from moribund orange-spotted grouper larvae collected in Hainan, China [44]. MSRV was isolated from California Bass larvae collected in Guangdong, China.

### Construction of recombinant plasmid

Total RNAs were extracted from GB cells, and cDNAs were synthesized using a reverse transcription kit. The full-length cDNA sequence of EcZNFX1 was obtained by PCR using specific primers. Codon optimization of the *Ecznfx1* sequence was performed according to mammalian codon usage preferences and cloned into pN3-HA plasmid. This process was completed by Guangzhou Ruibio Biotechnology Co., LTD. Mutants of EcZNFX1 were generated by PCR using specific primers based on the EcZNFX1-HA recombinant plasmid. The pRL-CMV plasmid was purchased from Beyotime Company. The plasmids EcMDA5-GFP, EcMAVS-GFP, EcTBK1-GFP, EcIRF3-GFP, EcIRF7-GFP, EcNF-κB-GFP, FHM-RGI-I-GFP, SSN-1-RIG-I-GFP, EcSTAT1a-flag, EcIFNh-HA and the firefly luciferase reporter vectors containing the promoters of EcZNFX1 (pGL3-EcZNFX1), EcIFNh (pGL3-EcIFNh), EcIFNd (pGL3-EcIFNd) and EcIFIT1 (pGL3-EcIFIT1) were constructed in our laboratory. The primer sequences are listed in Table 1.

**Table 1:** Primers used for cloning.

Name	Sequence (5’-3’)
ZNFX1-HA-F	GAGCTCATGGATAGGCTGACACTG
ZNFX1-HA-R	GAATTCGAAATGTGCAGCTGGTCAA
ZNFX1-N-F	CGCTTTCACAGAGGAGTCCAGATTCAATTCTGCAGTCGAC
ZNFX1-N-R	CCGCGGTACCGTCGACTGCAGAATTGAATCTGGACTCCTC
ZNFX1-∆N-F	GCTACCGGACTCAGATCTCGAGCTCAAGCTGGCTAGGATC
ZNFX1-∆N-R	GGTCTTTCTCGATCCTAGCCAGCTTGAGATCTGAGTCCGG
ZNFX1-P-loop-F	TCAGATCTCGAGCTCAAGCTTAAGCTGGCTAGGATCGAGAAAGA
ZNFX1-P-loop-R	GTACCGCGGTACCTTGAATTCGCACAGGGTCAGGGCTTTTC
ZNFX1-∆P-loop-F	CGCTTTCACAGAGGAGTCCAGATTCTGTCAGAACCACCCA
ZNFX1-∆P-loop-R	CCTGCCTGTCTGGGTGGTTCTGACAGAATCTGGACTCCTC
ZNFX1-ZF-F	GCTACCGGACTCAGATCTCGAGCTCTGCGGACACGTGTGT
ZNFX1-ZF-R	ACACCCTGGTACACACGTGTCCGCAGAGCTCGAGATCTGA
ZNFX1-∆ZF-F	AAAGCCTTGTGAGTTCAGACTGAAACTGCACCCTGCTTGG
ZNFX1-∆ZF-R	TAGCCTCGCTCCAAGCAGGGTGCAGTTTCAGTCTGAACTC
ZNFX1 promoter-F	AGAACATTTCTCTATCGATAGGTACCACATTTGCGGTGGTTGTGGA
ZNFX1 promoter-R	ACCAACAGTACCGGAATGCCAAGCTTGTGGAAACACCTGTGCTTTACCT

### Bioinformatics analysis

Phylogenetic tree construction was performed using the Neighbor-joining method in MEGA 7.0 software, with specific parameters as defined in previous studies [45]. For prediction of transcription factor binding sites in the promoter region, bioinformatics analysis was conducted using JASPAR (http://jaspar.genereg.net/) following the standard analytical workflow of the database.

### Antibodies and reagents

Anti-HA rabbit monoclonalantibody (1:3,000; 3724S; Cell Signaling), anti-GFP rabbit monoclonal antibody (1:3,000; 2956S; Cell Signaling), anti-flag rabbbit monoclonal antibody (1:3,000; 14793S; Cell Signaling), anti-pTBK1 rabbit monoclonal antibody (1:1,000; 5483S, Cell Signaling), β-actin mouse monoclonal antibody (1:3,000; 66009–1; Proteintech) and J2 anti-dsRNA mouse monoclonal antibody (1:1,000; 10010200; SCICONS) were used. Poly(I:C), including high molecular weight (HMW) and low molecular weight (LMW), poly(dA:dT) and biotin-poly(I:C)(HMW) were obtained from Invivogene.

### Cell transfection

Cells in 6-well plates were cultured for 12 h and transfected with 1.5 μg plasmids using High-efficiency Transfection Reagent/Superluminal (MIKX, 11231804–10) following the manufacturer’s instructions. For the dose-dependent assays, 1.0 μg, 2.5 μg, and 5.0 μg of plasmids were used respectively for transfection. For experiments related to antiviral function, cells were infected with the corresponding virus at 24 h post transient transfection.

### Analogue stimulation and virus infection

Cells were seeded into six-well plates and cultured for 12 h. For stimulation assays, cells were treated with poly(I:C)(HMW) (5 µg/mL), poly(I:C)(LMW) (5 µg/mL), or poly(dA:dT) (5 µg/mL) for 6 h. Healthy orange-spotted grouper were intraperitoneally injected with poly(I:C)(LMW) (10.0 μg/g) and poly(I:C)(HMW) (10.0 μg/g) or equal volume PBS for 3, 6, 12, and 24 h, to detected the transcription levels of EcZNFX1 in head kidneys. For temporal expression analysis of EcZNFX1, cells were infected with OGNNV (MOI = 2) or MSRV (MOI = 0.5) in DMEM containing 2% serum for 2 h, then washed with 1 × PBS and incubated in fresh DMEM with 2% serum for indicated time points. Additionally, cells were transfected with plasmids encoding CP-EGFP, B1-EGFP, B2-EGFP and ProA-EGFP, or treated with virus-like particle (VLP) protein (1 µg/mL) for 36 h. All collected samples were analyzed by quantitative reverse transcript-PCR (qRT-PCR) to determine mRNA levels.

### RNA interference

SiRNAs targeting EcZNFX1, EcMDA5 and FHM-RIG-I were designed and synthesized by RiboBio Co., Ltd. (Guangzhou, China). GB cells or GS cells were seeded into 6-well plates and transfected with 60-pmol siRNA for 24 h and challenged with OGNNV. FHM cells were seeded into 6-well plates and transfected with 60-pmol siRNA for 24 h and challenged with poly(I:C). All siRNA transfections were performed using Lipo8000 Transfection Reagent (Beyotime, C0533) following the manufacturer’s instructions (Table 2). Cells were harvested for RNA extraction followed by qRT-PCR. All the sequences of siRNAs are as follows:

**Table 2:** Sequences of siRNAs used in this study.

Name	Sequence (5’-3’)
siEcZNFX1–1	CGAACUCCAAUACGUAAGA
	UCUUACGUAUUGGAGUUCG
siEcZNFX1–2	GCAGACAUAAGAGGACAAA
	UUUGUCCUCUUAUGUCUGC
siEcZNFX1–3	GGAACUAGACGACAAGCUU
	AAGCUUGUCGUCUAGUUCC
siFHM-RIG-I-1	GCAGAGAUCUCUACAACUA
	UAGUUGUAGAGAUCUCUGC
siFHM-RIG-I-2	GCAGAAGUCUCAUCACAUC
	GAUGUGAUGAGACUUCUGC
siFHM-RIG-I-3	GAACAACAGUACAAGCUUU
	AAAGCUUGUACUGUUGUUC
siEcMDA5–1	GGAGCACAUACUGCGCAUU
	AAUGCGCAGUAUGUGCUCC
siEcMDA5–2	GAACCUGGCUCUCAGAAUU
	AAUUCUGAGAGCCAGGUUC
siEcMDA5–3	GCAUUGACAUGAUGAACAA
	UUGUUCAUCAUGUCAAUGC

### qRT-PCR

qRT-PCR analysis was performed with 2 × Polarsignal qPCR mix (MIKX, China, MKG800) on a Light Cycler480 instrument (Roche Diagnostics, Switzerland). The primers for qRT-PCR were designed using Primer premier software v5.0 program (Table 3). Each PCR assay was performed in triplicate. Gene expression levels were normalized to β-actin using the 2^−ΔΔCt^ method.

**Table 3:** Primers used for qRT-PCR.

Name	Sequence (5’-3’)
Q-CP-F	ATGGTGGGAAAGCAGAACAGT
Q-CP-R	ACAGGAGTATCAGCCGACCAG
Q-RdRp-F	GTGTCCGGAGAGGTTAAGGATG
Q-RdRp-R	CTTGAATTGATCAACGGTGAACA
Q-SCRV-G-140/187-F	AGCAGCATCACCAGCCACAT
Q-SCRV-G-187-R	CTCGTCCGTCGCTTGACTCA
Q-EcActin-F	TACGAGCTGCCTGACGGACA
Q-EcActin-R	GGCTGTGATCTCCTTCTGCA
Q-EcIFNh-F	TGTCCTTGGCTGCGATTGG
Q-EcIFNh-R	TTTGCTTGGAAAGGGAACTG
Q-EcIFIT1-F	ATTTGGCAGAGGAGGCT
Q-EcIFIT1-R	CTTTGCTTTGGGCGACT
Q-EcISG15-F	CCTATGACATCAAAGCTGACGAGAC
Q-EcISG15-R	GTGCTGTTGGCAGTGACGTTGTAGT
Q-EcMxI-F	CGAAAGTACCGTGGACGAGAA
Q-EcMxI-R	TGTTTGATCTGCTCCTTGACCAT
Q-EcZNFX1o-F	AGAACGGAAACGGCAACGA
Q-EcZNFX1o-R	TCGCCTCTAGGTCCGATGTT
Q-EcZNFX-F	TGTCACCAGGGACGGTATCA
Q-EcZNFX-R	CACACGGCTTCATACACTTACTG
Q-EcMDA5-F	ACCTGGCTCTCAGAATTACGAACA
Q-EcMDA5-R	TCTGCTCCTGGTGGTATTCGTTC
Q-EcIL-1β-F	AACCTCATCATCGCCACACA
Q-EcIL-1β-R	AGTTGCCTCACAACCGAACAC
Q-FHM-RIG-I-F	AACATCGAGCATCTGGCGAA
Q-FHM-RIG-I-R	CTGCAGCTCTTCTGAACCGA
Q-FHM-Actin-F	GGGCACCTGAACCTCTCATT
Q-FHM-Actin-R	CTGCTATGTGGCTCTTGACTTTG
Q-FHM-IFN-F	AAAACTCAAATGTGGACGTA
Q-FHM-IFN-R	GATAGTTTCCACCCATTTCCT
Q-FHM-ISG15-F	TAATGCCACAGTCGGTGAA
Q-FHM-ISG15-R	AGGTCCAGTGTTAGTGATGAGC

### Co-immunoprecipitation and Western blotting

HEK 293T cells or GS cells were co-transfected with the indicated plasmids. At 36 h post-transfection, the cells were washed with cold 1 × PBS and lysed for 30 min at 4°C using cell lysis buffer (Beyotime, China, P0013) supplemented with protease inhibitor cocktail (Sigma-Aldrich, P8340-1ML). The lysates were then centrifuged to remove cellular debris, and the supernatants were used for co-immunoprecipitation (Co-IP) assays following the established protocol. The supernatant was incubated with 20 µL of anti-HA magnetic beads for 1–2 h. The bead-protein-antibody complex was washed five times with IP wash buffer on a magnetic shelf. The total proteins and the bead-protein-antibody complex were then boiled in SDS-PAGE Protein Loading Buffer for 10 min, resolved by SDS-PAGE, and transferred to PVDF membranes (Merck Millipore). Membranes were blocked for 1 h at room temperature with 5% skimmed milk powder dissolved in Tris-buffered saline containing 0.1% Tween-20 (TBST). The membranes were then incubated with the indicated primary antibodies, followed by the corresponding HRP-conjugated secondary antibodies, both diluted in TBST. Chemiluminescent signals were detected using the 5200 Chemiluminescence Imaging System (Tanon). Protein levels were quantified using ImageJ 1.53 (National Institutes of Health).

### In vitro RNA pulldown

For poly(I:C) pulldown assays, unlabeled poly(I:C) and biotinylated poly(I:C) (each at a final concentration of 1 µM) were incubated with Dynabeads M-280 Streptavidin (Thermo Fisher Scientific, 11205D) for 30 min at 4°C in binding buffer (50 mM Tris-HCl, pH 7.5, 150 mM NaCl, 0.5 mM EDTA, 10% glycerol, 1 mM 2-mercaptoethanol, and 0.5% Nonidet P-40, pH 7.5). After washing with binding buffer three times, the beads were incubated with whole-cell lysates from HEK 293T cells transiently transfected with the indicated expression plasmids for 1 h at 4°C. The beads were then washed four times with wash buffer (50 mM Tris-HCl, pH 7.5, 1 M NaCl, 0.5 mM EDTA, 10% glycerol, 0.5 mM EDTA, 1 mM 2-mercaptoethanol, and 1% Nonidet P-40). Bound proteins were eluted by boiling in SDS-PAGE sample buffer for 10 min at 100°C.

### RNA immunoprecipitation (RIP)

GB cells were transiently transfected with the indicated HA-tagged plasmids for 24 h, followed by OGNNV infection for an additional 24 h. Cells were then collected and lysed in immunoprecipitation lysis buffer supplemented with RNase inhibitor (ThermoFisher, EO0382) and protease inhibitor cocktail. Protein A/G beads were incubated with anti-HA rabbit monoclonal antibody or normal rabbit IgG in RIP buffer (25 mM Tris-HCl, pH 7.5, 150 mM KCl, 0.5 mM DTT, 20 mM EDTA, 1% NP-40) for 30 min. The beads were subsequently added to the cell lysate with an equal volume of 2 × RIP buffer, and incubated for 5 h at 4°C. The bead-bound immunoprecipitates were washed five times with RIP wash buffer (25 mM Tris-HCl, pH 7.5, 150 mM KCl, 0.5 mM DTT, 5 mM EDTA, 0.5% NP-40) and split into two samples for protein or RNA extraction. Proteins were extracted for Western blot analysis, while RNA was extracted using Trizol reagent for subsequent real-time PCR analysis targeting OGNNV RNA.

### Immunofluorescence staining and confocal microscopy observation

GB, GS or HEK 293T cells were seeded on glass coverslips in 24-well plates, then transiently transfected with 0.5 μg the indicated plasmids. For the co-localization experiment of EcZNFX1 and OGNNV dsRNA, the cells were subsequently infected with OGNNV for 24 h. The transfected cells were harvested for Immunofluorescence assay as previously described [36].

### Luciferase reporter gene assay

GB or GS cells were plated in 12-well plates. Cells in each well were transiently transfected with 20 ng pRL-CMV plasmid, 100 ng pGL3-EcZNFX1/pGL3-EcIFNh/pGL3-EcIFNd or pGL3-EcIFIT1 and 1 μg indicated plasmids. At 24 h post-transfection, cells were infected with OGNNV for an additional 24 h. Luciferase activities were then measured using the Dual-Luciferase Reporter Assay System (Promega) according to the manufacturer’s instructions. Transfection efficiency was normalized by dividing Firefly luciferase activity by Renilla luciferase activity. Each experiment was performed in triplicate.

### Pyroptosis analyses

GB cells were transiently transfected with the indicated plasmids for 12 h and infected with OGNNV for 36 h. Cells undergoing Pyroptosis was determined using a FAM-FLICA detection kit (ImmunoChemistry, ICT-97) according to the manufacturer’s instructions.

### Statistical analyses

All statistical analyses were performed using GraphPad Prism 10.1.2 (GraphPad Software). Data are presented as means ± standard deviation (SD) from at least three independent experiments. A bilateral unpaired Student t-test or one-way ANOVA was used to calculate the statistical differences between the two groups. Significance levels are indicated as follows: *P < 0.05, **P < 0.01, ***P < 0.001, and ns for non-significant differences.

## Supporting information

S1 Fig Sequence alignment of EcZNFX1 and ZNFX1 from *Sander lucioperca*, *Dendropsophus ebraccatus* and *Homo sapiens.* Identical and similar amino acid residues are shaded in blue and light blue, respectively.
(TIF)

S2 Fig OGNNV infection and poly(I:C) stimulation upregulates the expression of EcZNFX1. (A) GB cells were stimulated with poly(I:C)(HMW) or poly(dA:dT) for 0 h, 3 h, 6 h and 12 h. The mRNA levels of EcZNFX1 were analysed by qRT-PCR. (B) The mRNA levels of RDRP in OGNNV-infected GB cells were detected by qRT-PCR analysis. (C) The mRNA levels of EcZNFX1 in brain and spleen of healthy or OGNNV-infected orange-spotted grouper were detected by qRT-PCR analysis. The data are shown as the means ± SDs. Statistical differences were determined by Student t-test. *P < 0.05, **P < 0.01, ***P < 0.001, ns, no significance.
(TIF)

S3 Fig Validation of EcZNFX1, FHM-RIG-I and EcMDA5 Knockdown Efficiency. The mRNA levels of EcZNFX1, FHM-RIG-I and EcMDA5 in GB and FHM cells transfected with siRNAs targeting control (siNC), EcZNFX1 (siEcZNFX1), FHM-RIG-I (siFHM-RIG-I) and EcMDA5 (siEcMDA5) were detected by qRT-PCR. The data are shown as the means ± SDs. Statistical differences were determined by one-way ANOVA. *P < 0.05, **P < 0.01, ***P < 0.001, ns, no significance.
(TIF)

S4 Fig The mRNA levels of EcIL-1β in OGNNV-infected GS cells were detected by qRT-PCR analysis. The data are shown as the means ± SDs. Statistical differences were determined by one-way ANOVA. *P < 0.05, **P < 0.01, ***P < 0.001, ns, no significance.
(TIF)

S1 Dataset The values used to build graphs in this study. (ZIP)
