# Supplementary figures and images for: ZNFX1 functions as a compensatory dsRNA recognition receptor to exert antiviral effect in orange-spotted grouper
Source: PLoS Pathog. 2025 Oct 29;21(10):e1013652. doi: 10.1371/journal.ppat.1013652 (PMC12585102; doi:10.1371/journal.ppat.1013652)

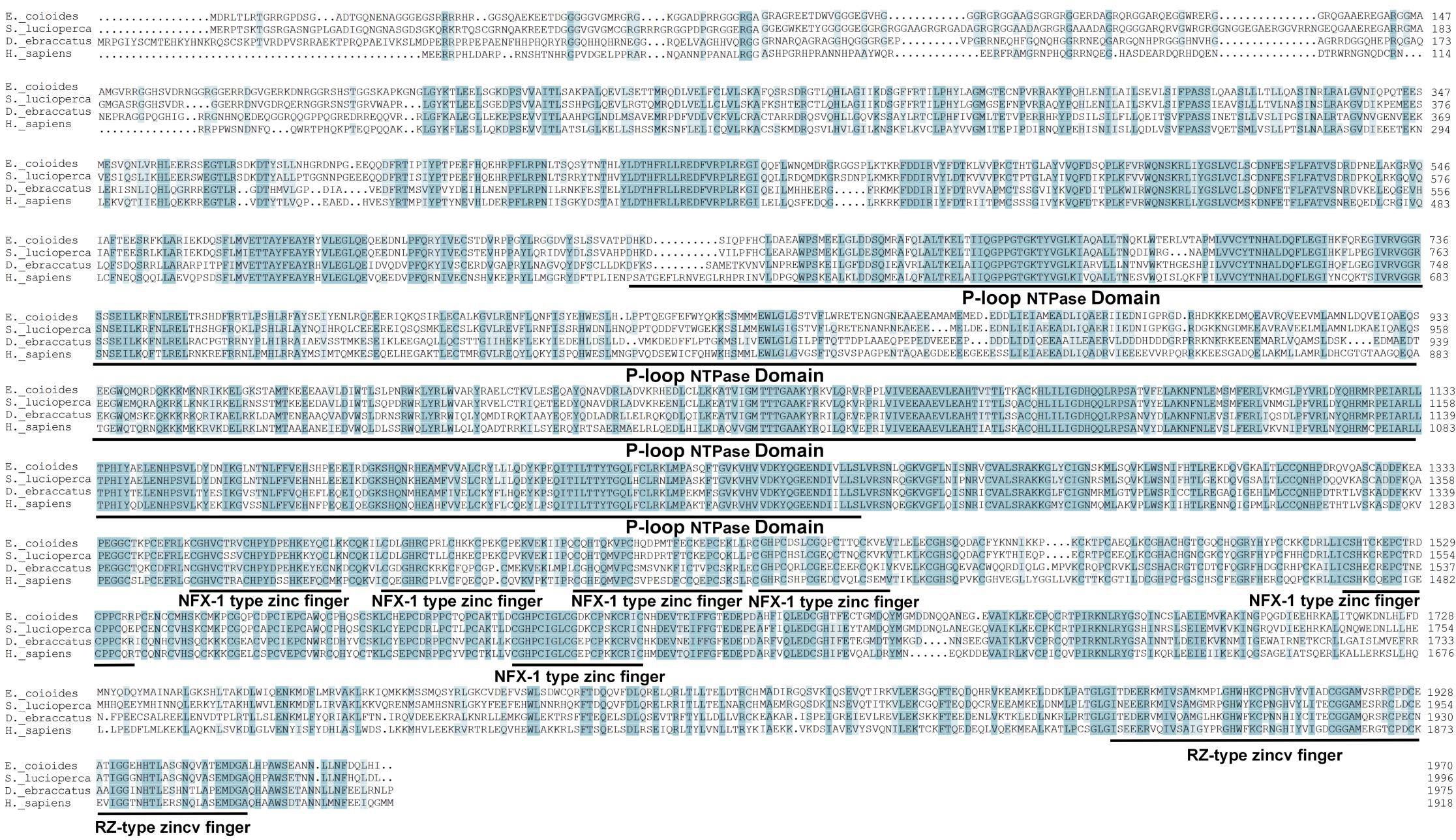

Supplement: S1 Fig — Identical and similar amino acid residues are shaded in blue and light blue, respectively. (TIF) [file ppat.1013652.s001.tif]

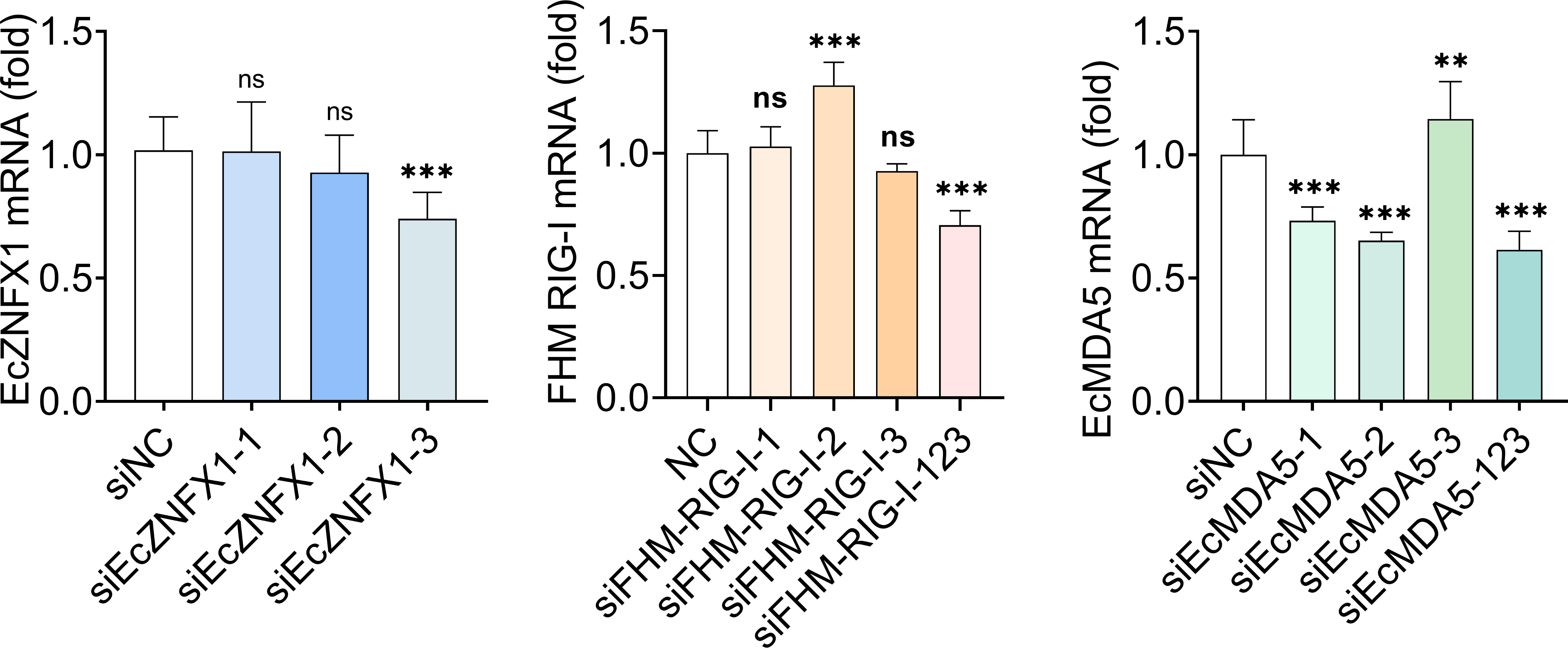

Supplement: S3 Fig — The mRNA levels of EcZNFX1, FHM-RIG-I and EcMDA5 in GB and FHM cells transfected with siRNAs targeting control (siNC), EcZNFX1 (siEcZNFX1), FHM-RIG-I (siFHM-RIG-I) and EcMDA5 (siEcMDA5) were detected by qRT-PCR. The data are shown as the means ± SDs. Statistical differences were determined by one-way ANOVA. *P < 0.05, **P < 0.01, ***P < 0.001, ns, no significance. (TIF) [file ppat.1013652.s003.tif]

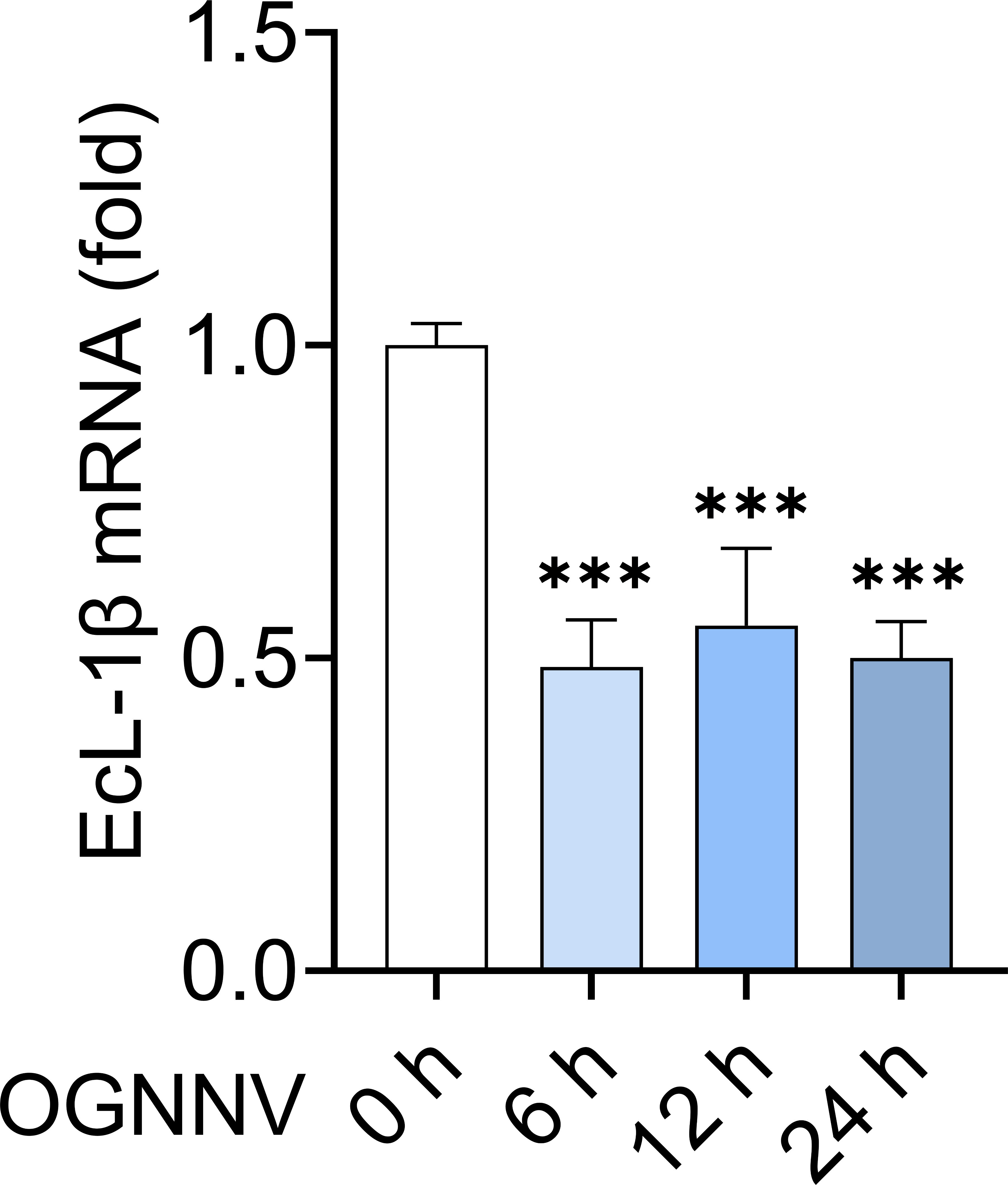

Supplement: S4 Fig — The data are shown as the means ± SDs. Statistical differences were determined by one-way ANOVA. *P < 0.05, **P < 0.01, ***P < 0.001, ns, no significance. (TIF) [file ppat.1013652.s004.tif]
