# Supplementary material for: ZNFX1 functions as a compensatory dsRNA recognition receptor to exert antiviral effect in orange-spotted grouper
Source: PLoS Pathog. 2025 Oct 29;21(10):e1013652. doi: 10.1371/journal.ppat.1013652 (PMC12585102; doi:10.1371/journal.ppat.1013652)
Supplement: S1 Dataset — (ZIP) [file ppat.1013652.s005.zip › DATA1/original WB image.pdf]

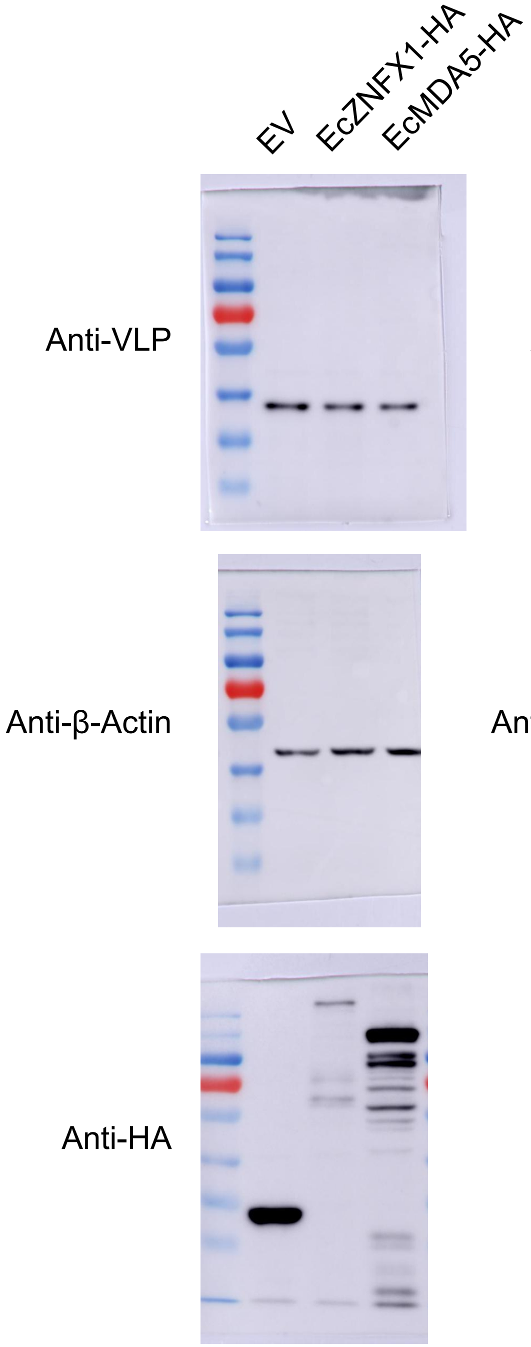

Figure 3. B

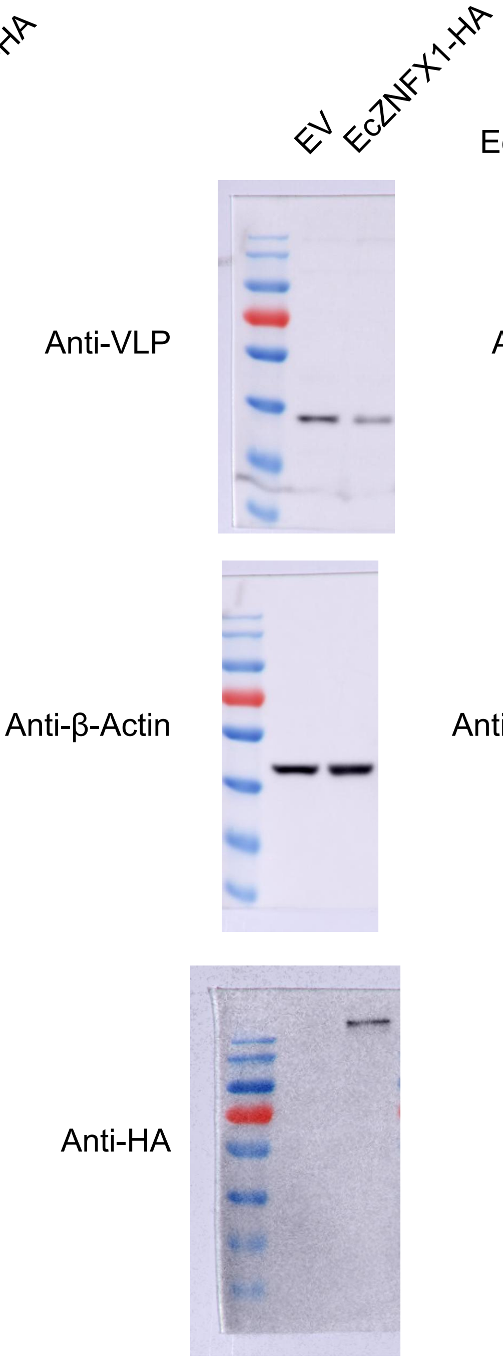

Figure 3. D

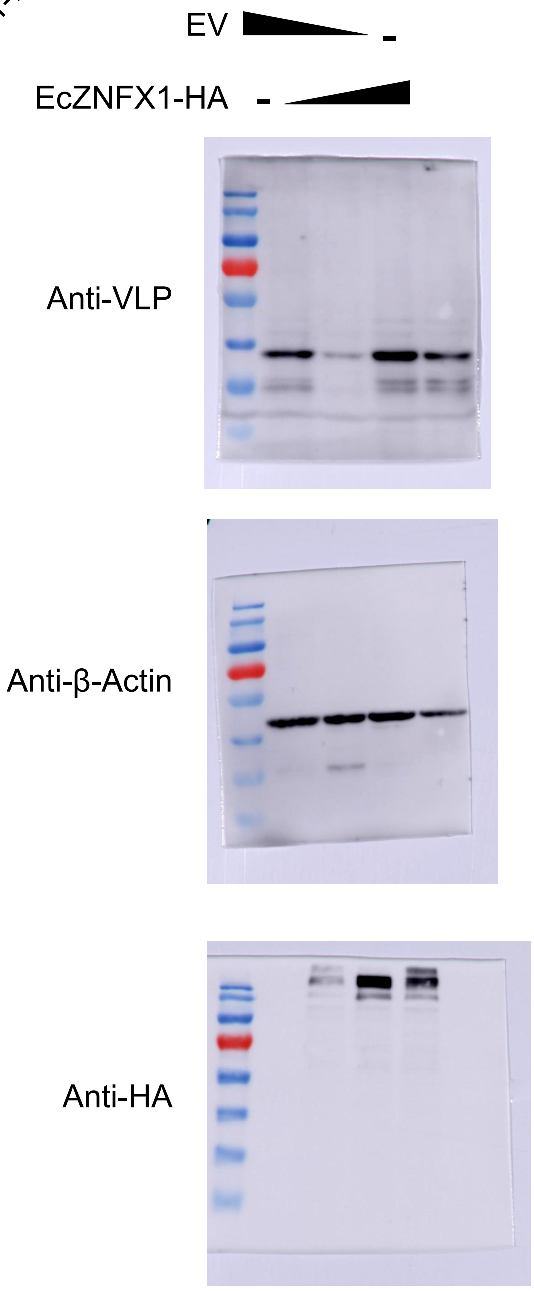

Figure 4. E

The original WB image involved in Fig 3. and Fig 4.

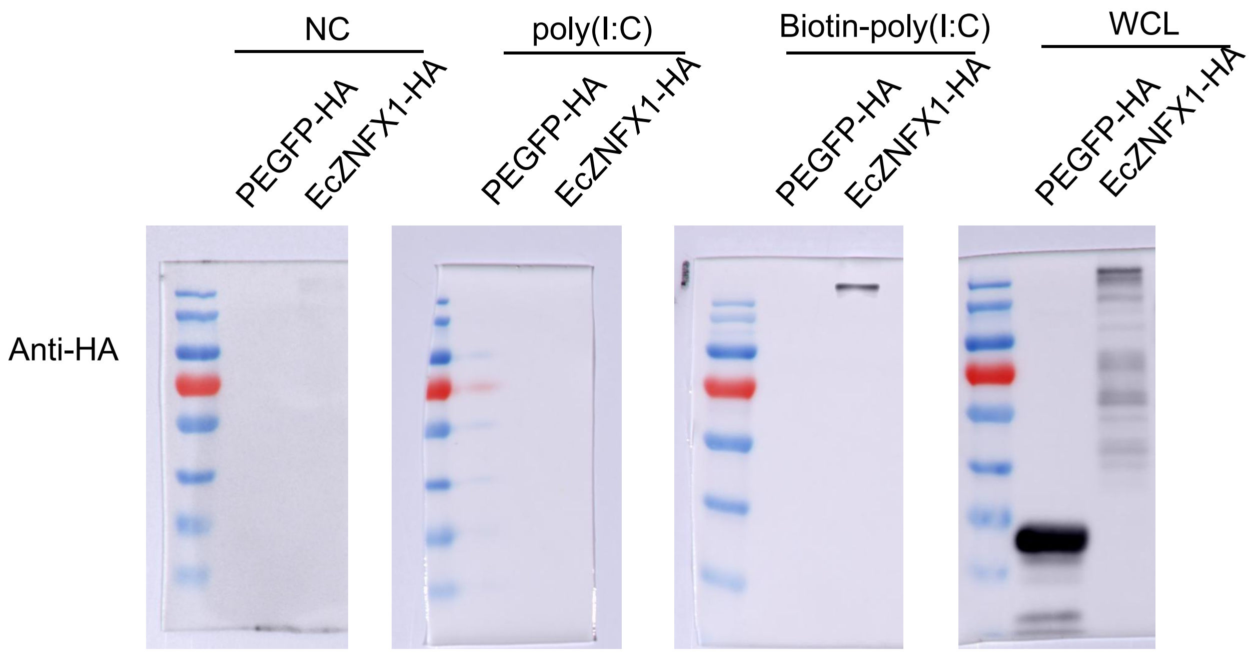

Figure 5. A

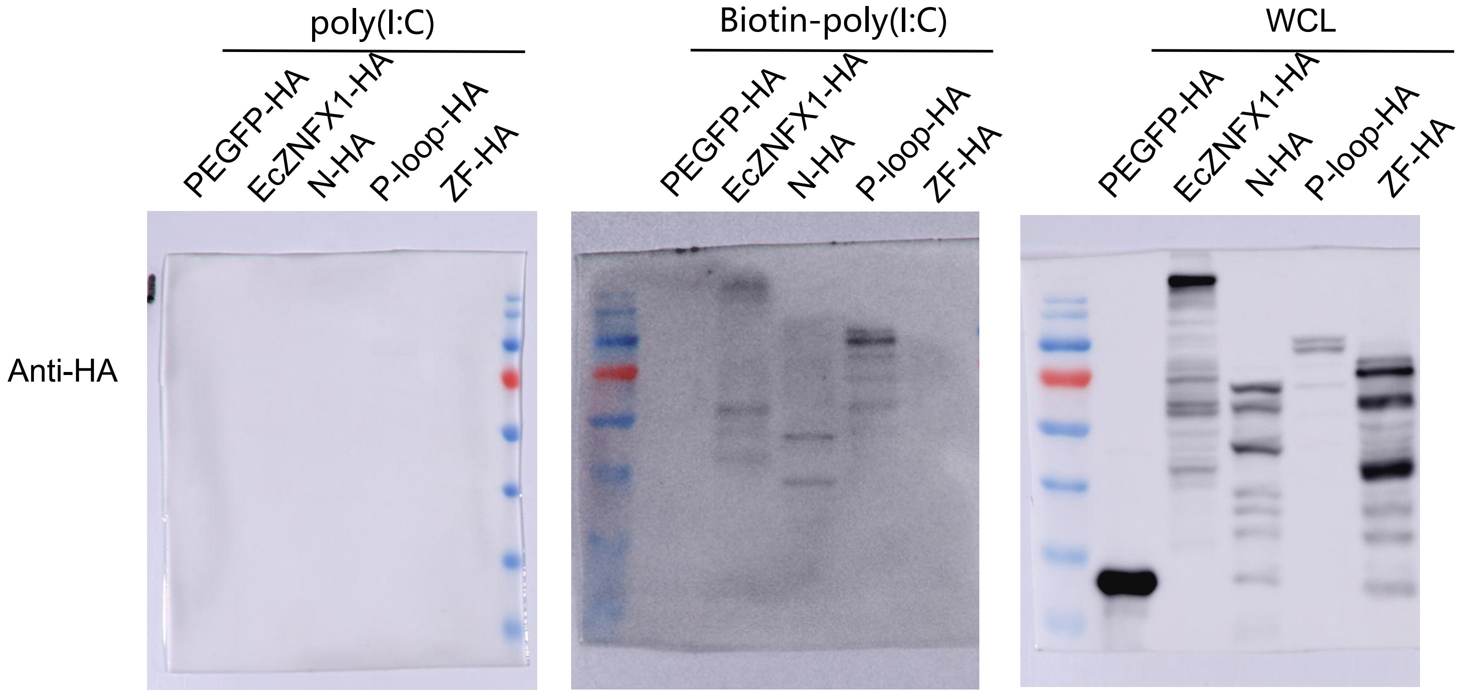

Figure 5. B

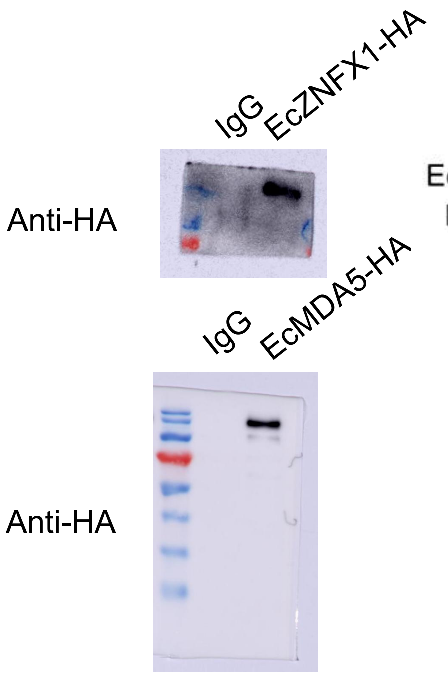

Figure 5. C

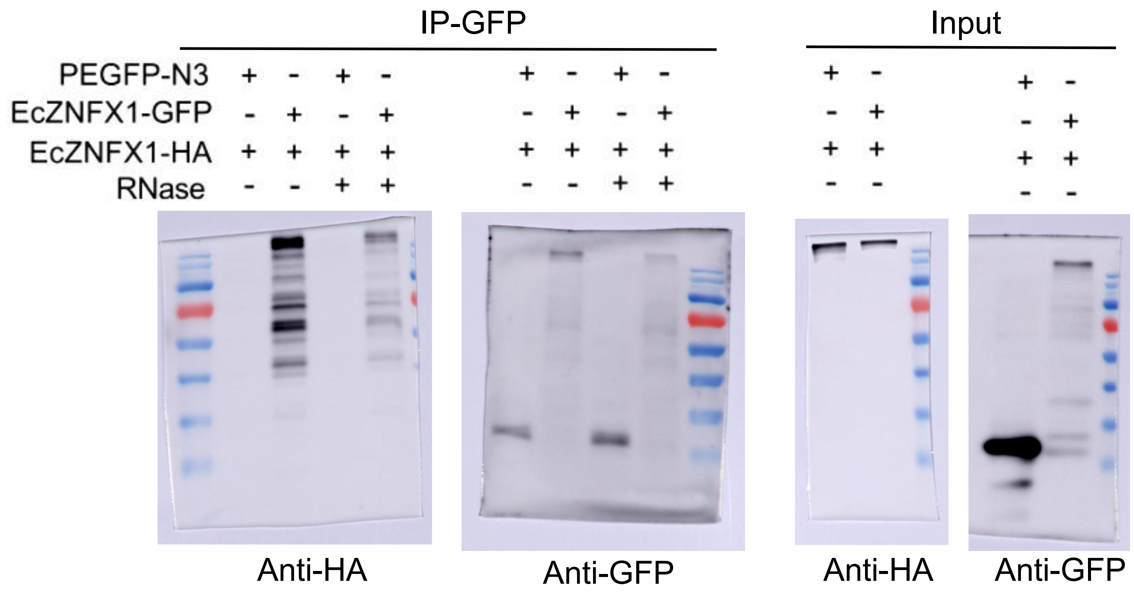

Figure 5. E

The original WB image involved in Fig 5.

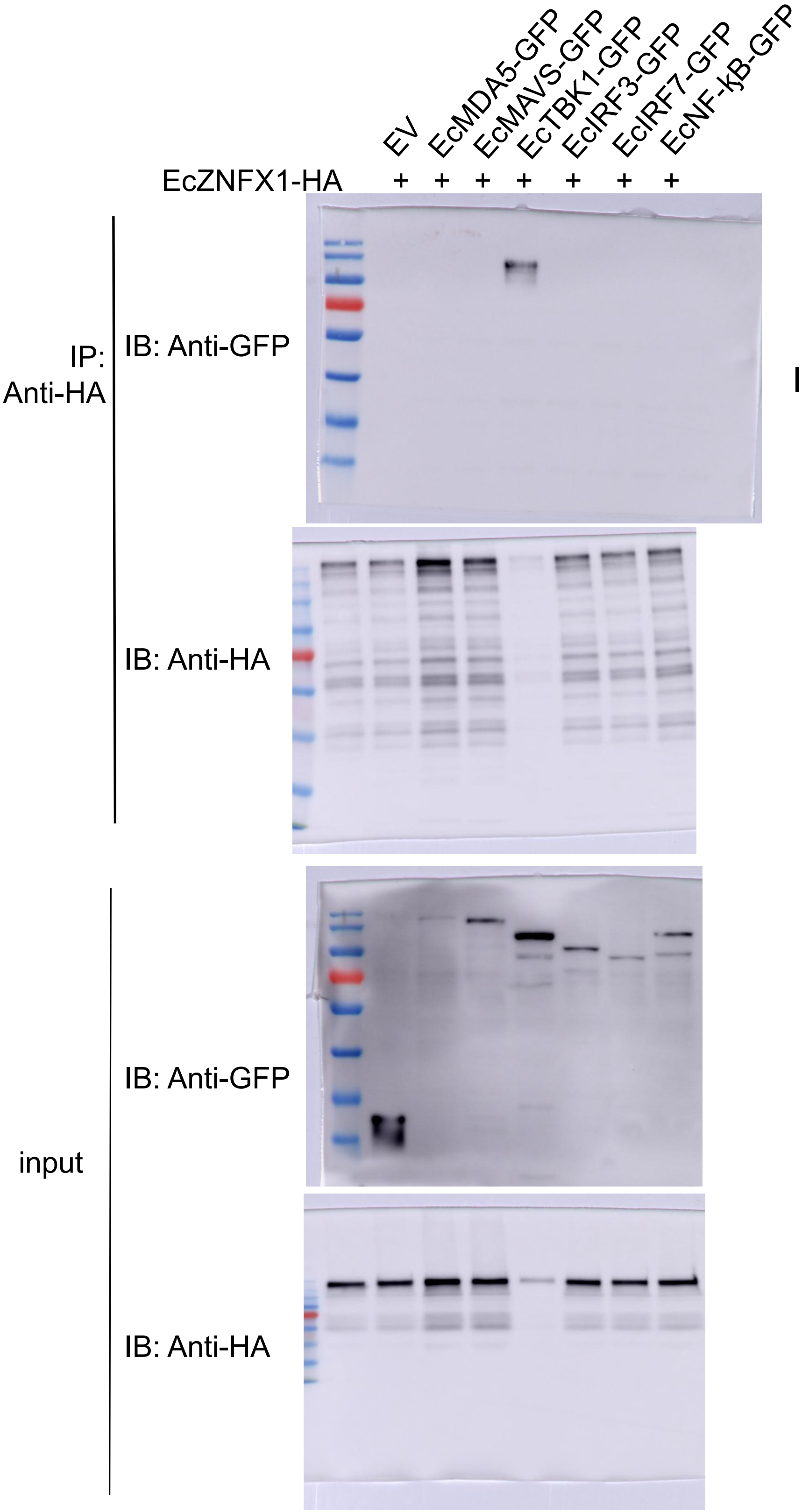

Figure 8. A

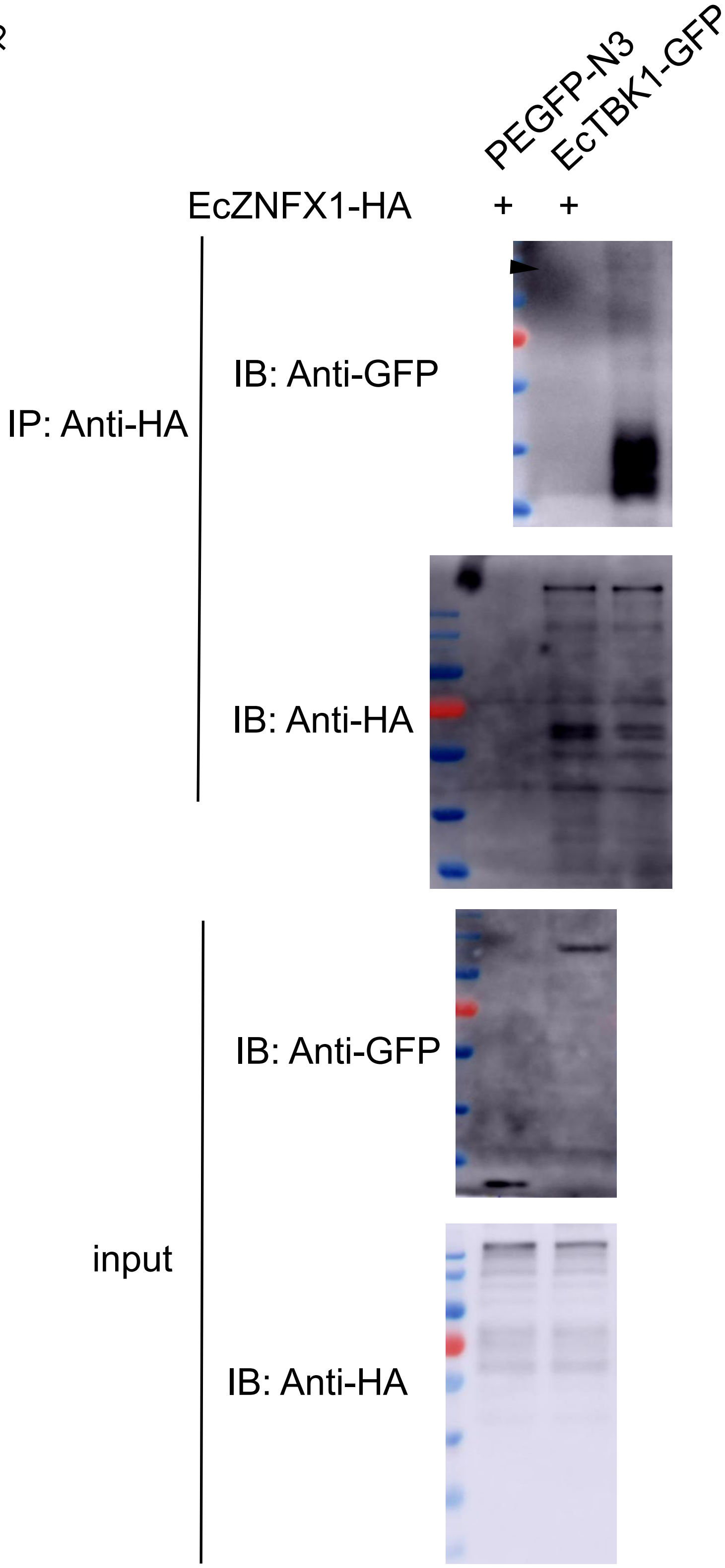

Figure 8. B

The original WB image involved in Fig 8.

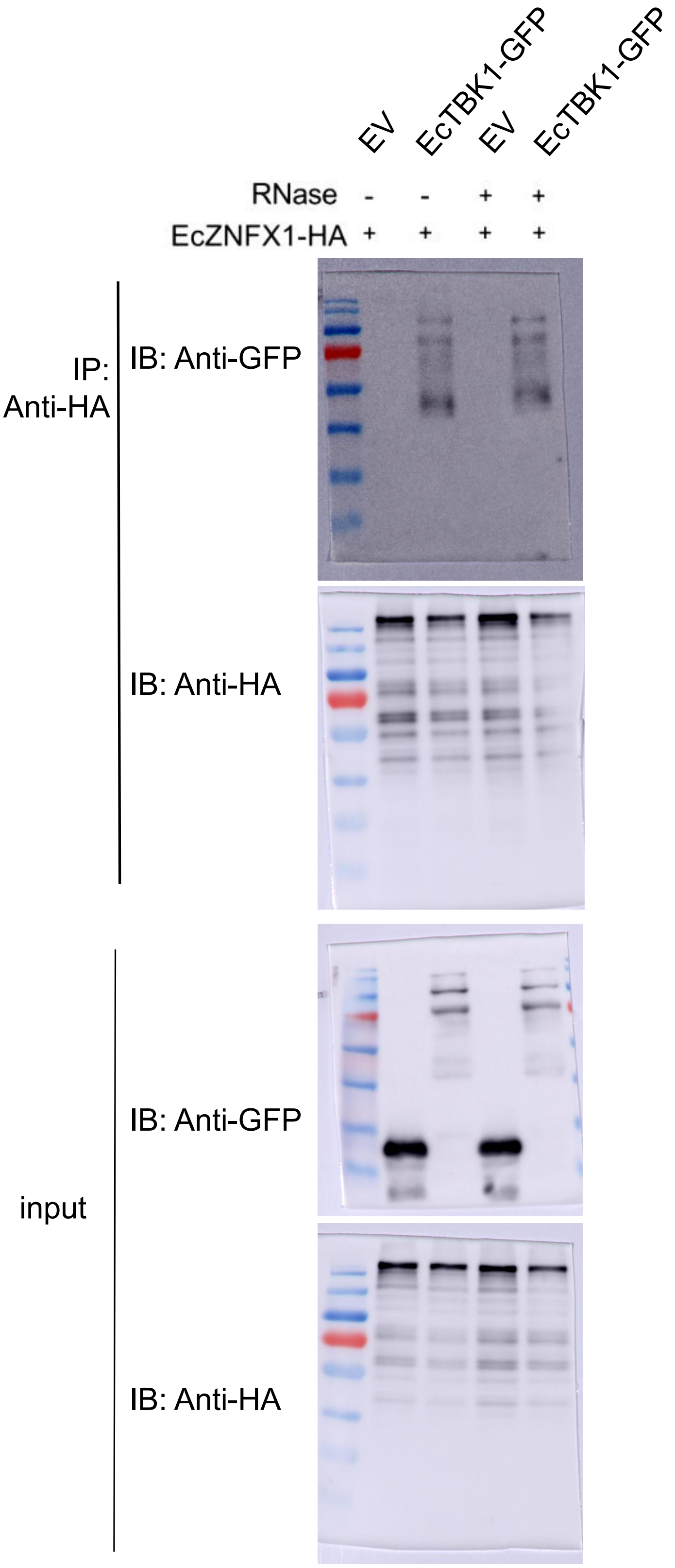

Figure 8. C

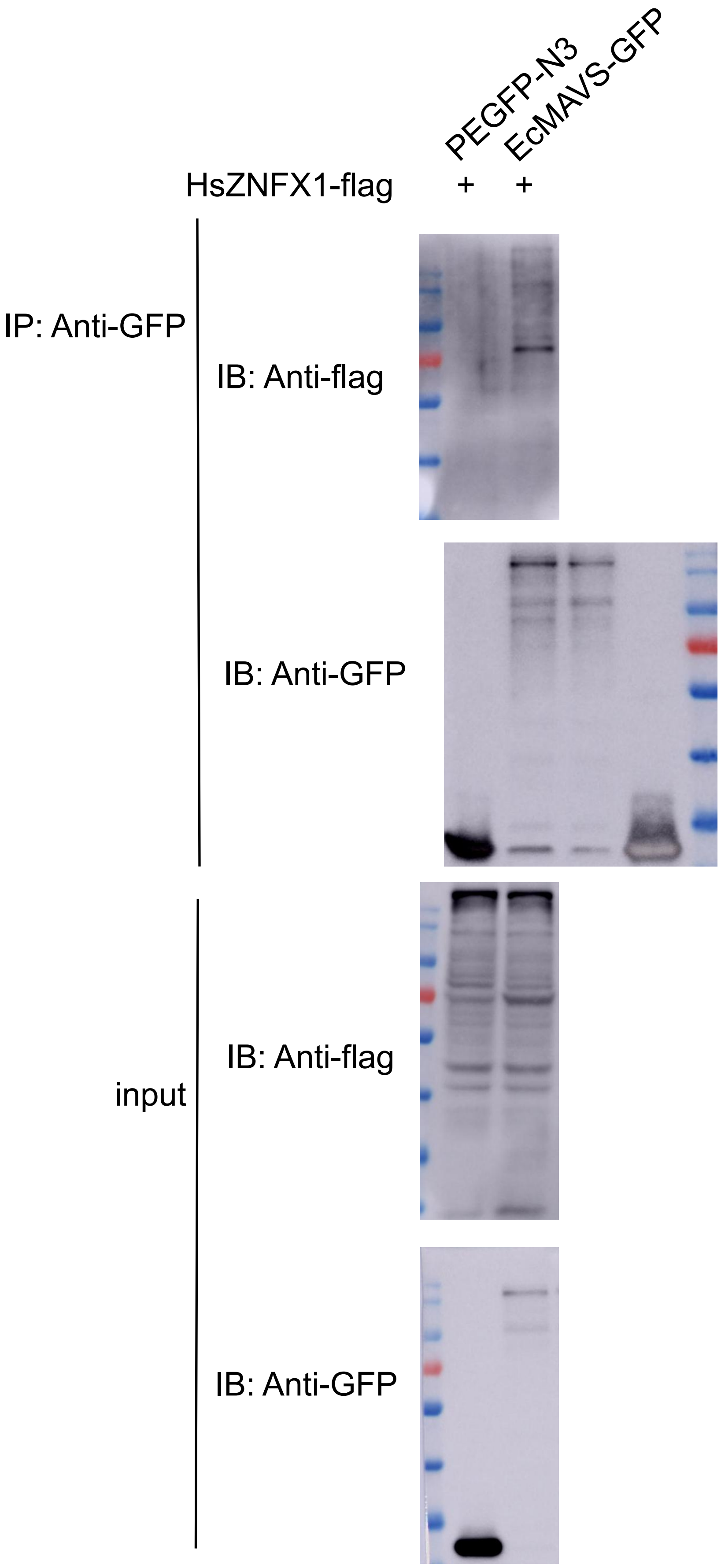

Figure 8. E

The original WB image involved in Fig 8.

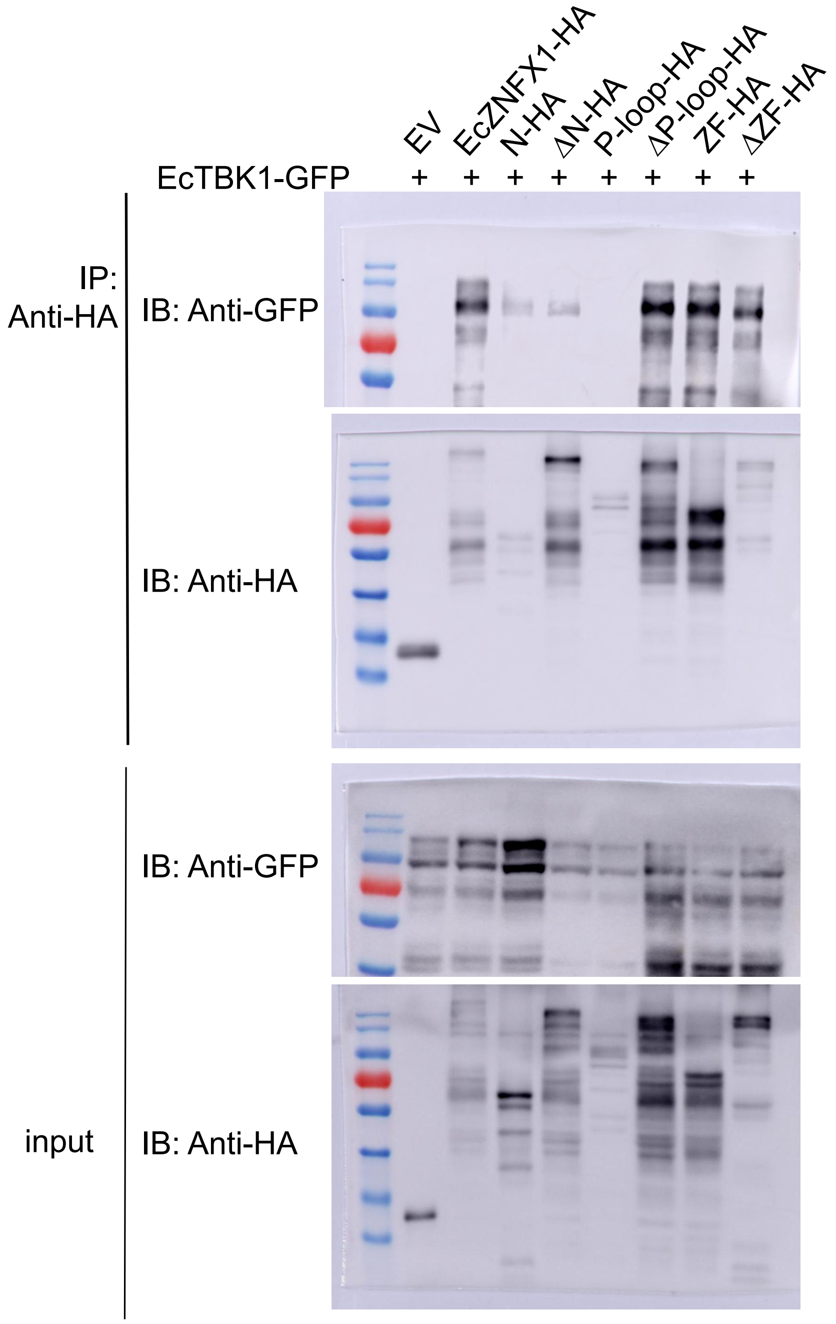

Figure 8. D

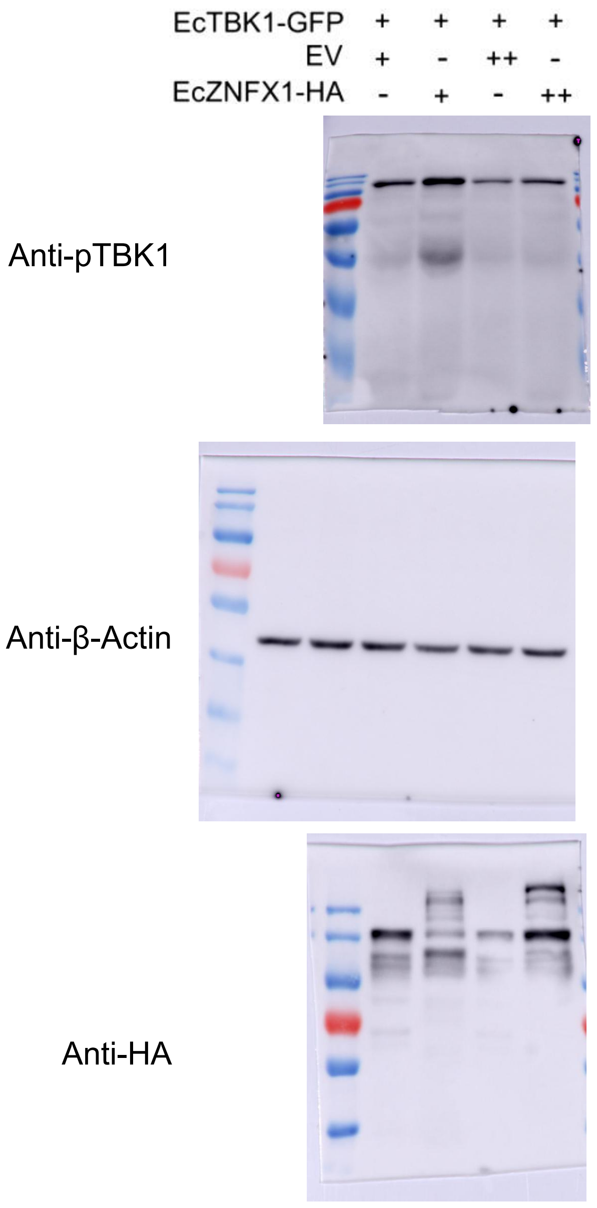

Figure 8. F

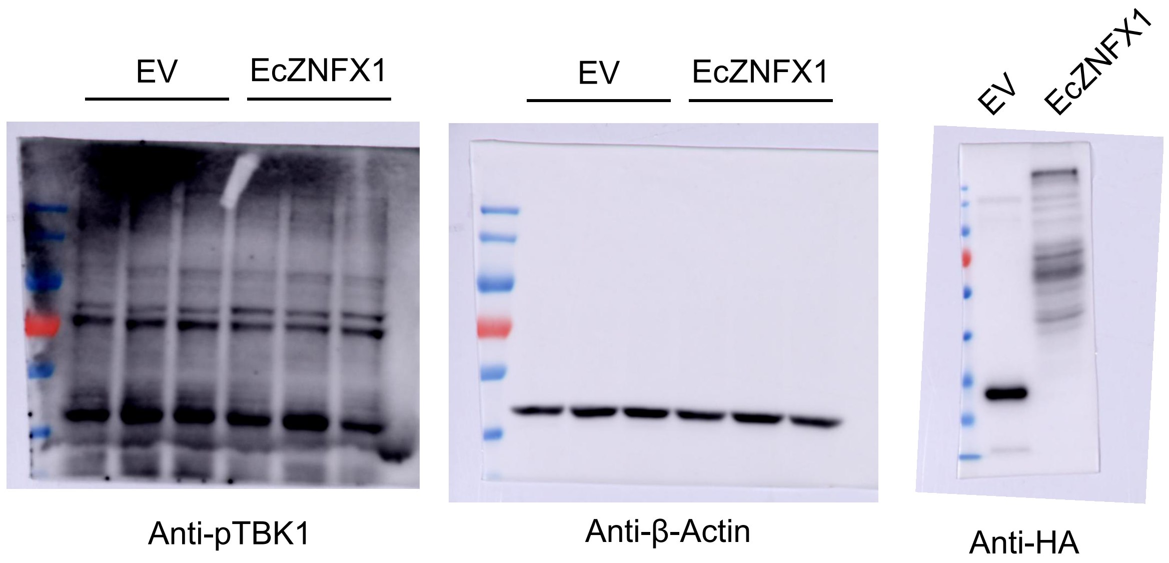

Figure 8. G

The original WB image involved in Fig 8.
